# Power Electronics Revolutionized: A Comprehensive Analysis of Emerging Wide and Ultrawide Bandgap Devices

**DOI:** 10.3390/mi14112045

**Published:** 2023-10-31

**Authors:** S M Sajjad Hossain Rafin, Roni Ahmed, Md. Asadul Haque, Md. Kamal Hossain, Md. Asikul Haque, Osama A. Mohammed

**Affiliations:** 1 Energy Systems Research Laboratory, Department of ECE, Florida International University, Miami, FL 33174, USA; srafi010@fiu.edu; 2 Department of ECE, Presidency University, Dhaka 1212, Bangladesh; roniahmed355@pu.edu.bd; 3 Department of EEE, Northern University Bangladesh, Dhaka 1230, Bangladesh; mdasadeee91@gmail.com (M.A.H.); kamalbijoy25@gmail.com (M.K.H.); asikul.haque.bd@gmail.com (M.A.H.)

**Keywords:** wide bandgap devices, ultrawide bandgap devices, silicon, silicon carbide, GaN, diamond, power semiconductor devices

## Abstract

This article provides a comprehensive review of wide and ultrawide bandgap power electronic semiconductor devices, comparing silicon (Si), silicon carbide (SiC), gallium nitride (GaN), and the emerging device diamond technology. Key parameters examined include bandgap, critical electric field, electron mobility, voltage/current ratings, switching frequency, and device packaging. The historical evolution of each material is traced from early research devices to current commercial offerings. Significant focus is given to SiC and GaN as they are now actively competing with Si devices in the market, enabled by their higher bandgaps. The paper details advancements in material growth, device architectures, reliability, and manufacturing that have allowed SiC and GaN adoption in electric vehicles, renewable energy, aerospace, and other applications requiring high power density, efficiency, and frequency operation. Performance enhancements over Si are quantified. However, the challenges associated with the advancements of these devices are also elaborately described: material availability, thermal management, gate drive design, electrical insulation, and electromagnetic interference. Alongside the cost reduction through improved manufacturing, material availability, thermal management, gate drive design, electrical insulation, and electromagnetic interference are critical hurdles of this technology. The review analyzes these issues and emerging solutions using advanced packaging, circuit integration, novel cooling techniques, and modeling. Overall, the manuscript provides a timely, rigorous examination of the state of the art in wide bandgap power semiconductors. It balances theoretical potential and practical limitations while assessing commercial readiness and mapping trajectories for further innovation. This article will benefit researchers and professionals advancing power electronic systems.

## 1. Introduction

In the era of power electronics, wide and ultrawide bandgap power electronic semiconductors have become a game-changing innovation. These cutting-edge materials, such as silicon carbide (SiC), gallium nitride (GaN), and diamond, perform better than conventional Si-based products. In recent years, significant improvements have been made in wide bandgap power electronic semiconductors regarding the materials’ caliber, device design, and production techniques. The creation of superior SiC and GaN substrates, advancements in crystal growth methods, and improved device production procedures have all been created by academics and business stakeholders. Wide bandgap devices are becoming more commercially viable due to these developments’ increased material performance, greater device yields, and lower production costs. Electronic switching devices are essentially used in power electronic converters to control electrical energy efficiently. Higher efficiency, greater power densities, and more integrated systems have always been the direction of power electronics technology development. Like many technologies, power semiconductor technology has been growing towards this constant progress. The development of diverse Si power devices over the past 50 years has been the main driver of the advancements [1].

Si is currently, by far, the most established semiconductor material used in power devices. However, due to its limitations, engineers and academics have made significant efforts to identify alternatives to Si-based power devices for greater performance. These devices are getting close to their material limits [2]. The introduction of power devices based on wide bandgap (WBG) materials such as SiC and GaN has been a revolutionary advancement. Utilizing these new wide bandgaps (WBGs) power semiconductor devices increases the efficiency of electric energy transformations, allowing for a more logical use of electric energy and a significant reduction in power converter size and robustness. SiC and GaN are excellent trade-offs between theoretical and practical properties among the possible semiconductor materials candidates. Moreover, these materials’ key advantages over Si include good performance across a wide temperature range, high dielectric strength, and high saturation drift velocity [3].

SiC is one of the most widely studied and commercially available wide bandgap materials. It possesses a bandgap energy of approximately 3.3 electron volts (eV), significantly higher than Si’s 1.1 eV. SiC-based power devices offer numerous advantages, including reduced conduction and switching losses, higher temperature tolerance, and increased efficiency. These properties make SiC devices well-suited for electric vehicles, renewable energy systems, industrial motor drives, and aerospace applications [4,5]. GaN is another prominent wide bandgap material recently gaining significant attention. GaN exhibits a bandgap energy of around 3.4 eV, similar to SiC. GaN-based power devices provide exceptional performance characteristics, including high breakdown voltages, fast switching speeds, and low on-resistance. These attributes make GaN devices ideal for applications requiring high-frequency operation, such as wireless power transfer, data centers, and radar systems.

It is widely known that, when running under reverse bias in the natural environment, Si power metal oxide field effect transistors (MOSFETs) are highly capable of single-event burnout (SEB) [6,7]. The parasitic bipolar transistor built into the design may turn on due to the transitory current created by intense heavy ion penetration through the device. Voltage ranges can vary up to 600 V regarding Si MOSFETs [8]. With the support of more than 600 V voltage applications, insulated gate bipolar transistors (IGBTs) have developed necessary applications. However, due to their maximum switching losses, IGBT devices have become low-efficient at high frequencies [9].

Over the past 20 years, a great deal of research has been carried out on SiC power devices, and many are currently in the market. In particular, SiC is utilized outside of semiconducting, including in ceramic plates, thin filament pyrometry, foundry crucibles, bulletproof jackets, and auto clutches. Because it may operate at greater temperatures, higher current densities, and higher blocking voltages because of the wider bandgap, higher thermal conductivity, and larger critical electric field [10,11,12,13], one of its earliest uses in electrical applications was as a lightning arrester in a high-voltage power system. Schottky diodes, MOSFETs, IGBTs, and power electronics are examples of SiC’s recent use in electronics. 

**Figure 1:**
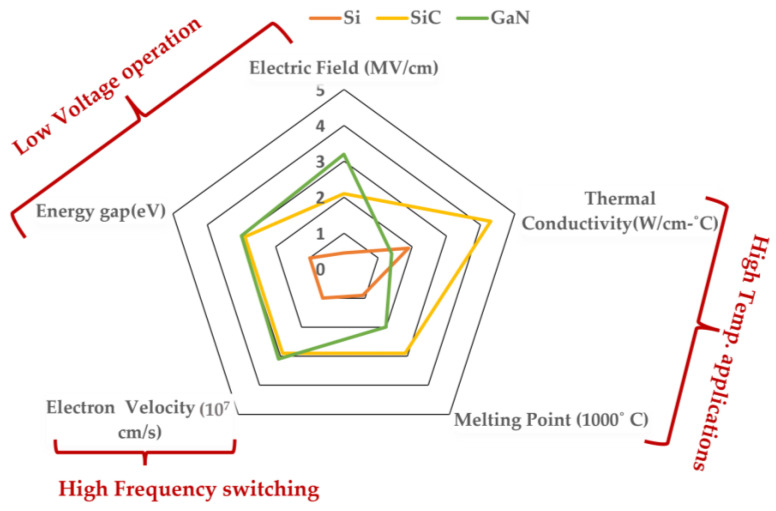
Summary of Si’s, SiC’s, and GaN’s relevant material properties [1].

Compared to SiC technology, GaN has a greater bandgap energy and higher electron mobility [14]. It has also progressed in the low- to medium-voltage, high-frequency sector. For high-frequency applications based on lateral transistors, GaN is more effective. Both materials can offer better performances than the Si devices on the market [15,16], but the many technological processes for transistor manufacture must be properly integrated. GaN-based Field Effect Transistors (FETs), also known as, GaN High Electron Mobility Transistor (HEMTs) can switch faster than Si power transistors. GaN HEMTs have a tiny physical growth, which enables the devices to be more energy-efficient and high voltage application while providing extra space for external components. The properties of Si, SiC, and GaN have been demonstrated in Figure 1.

SiC is the third hardest substance on earth and is known to be as hard as a single crystal. The SiC energy gap ranges from 2 to 3.3 eV depending on the polytype crystal structure. SiCs are the ideal choice among commercially available WBG devices due to their greater power rating, quicker switching frequency, much lower switching losses, and capacity to handle higher junction temperatures than Si-based devices. Another promising wide bandgap semiconductor material GaN [16,17,18,19] is an example of the third generation of semiconductor materials, with a wider bandgap (bandgap width of more than 3.4 eV), high critical breakdown electric field, high anti-radiation ability, and rapid electron saturation velocity. GaN material has a wide range of applications and is one of the most efficient ways to save energy and reduce consumption worldwide. It summarizes recent research on GaN technology, demonstrating the slow but steady development of a local GaN supply chain.

Wide bandgap power electronic semiconductors are now in the state of active research and development. Researchers are constantly investigating novel device architectures, packaging methods, and heat management strategies to improve further the performance, reliability, and efficiency of wide bandgap devices. These initiatives tackle issues including enhancing device dependability, cutting manufacturing costs, and boosting system-level integration. Wide bandgap power electronic semiconductor applications are also being expanded into new markets, including 5G wireless communications, the Internet of Things (IoT), and sophisticated medical equipment. Wide bandgap devices thrive in these applications because they need excellent power density, quick switching times, and high-frequency functioning.

Ultrawide bandgap (UWBG) semiconductors like diamond enable incredibly promising electrical gadgets. Diamond has a band gap of 5.5 eV broad, more than five times that of Si [17]. Diamond transistor devices can theoretically switch at frequencies over 100 GHz and function at temperatures higher than 600 °C. High-power diamond Schottky diodes for power electronics, diamond UV detectors for monitoring flames, and diamond radiation detectors for physics research are a few unusual applications. Diamond’s excellent heat conductivity and breakdown voltage enable incredibly small, effective power devices. The difficulties in doping diamonds to create trustworthy ohmic connections and the constraints in manufacturing large single-crystal diamond wafers are obstacles to developing diamond electronics. However, in situ-doped polycrystalline diamond films and diamond-on-Si techniques are advancing the field [17]. Diamond has the potential for orders of magnitude of improvements in power density, operating temperature, radiation hardness, and switching speed compared to traditional electronics. Deep space missions to power grid electronics could benefit from revolutionary applications if the diamond’s full potential is realized. Due to their high-efficiency power conversion, electric vehicles have longer driven ranges and require less time to charge. Utilizing wide bandgap technology, renewable energy systems like solar and wind power may maximize energy collecting and grid integration [18]. Moreover, their high-power density and enhanced thermal management capabilities are advantageous for aerospace and defense applications.

This manuscript first provides the background on power electronics and wide bandgap materials. After that, in separate sections for each material, it describes the properties, historical development, devices, applications, and difficulties of the major semiconductors: Si, SiC, GaN, and diamond. It then assesses recent advancements in IGBTs and MOSFETs and compares performance characteristics like bandgap, breakdown voltage, and switching frequency across the materials. Adoption trends and the current commercial environment are also covered. The conclusion summarizes the results and provides a forecast for future developments. The review is organized to present a thorough technology overview, evaluate the state of the art, and give strategic recommendations to researchers and industry professionals working on the future generation of high-efficiency power semiconductors.

The focus of this article is to provide an extensive review of wide and ultrawide bandgap power semiconductor devices. From the earliest device inventions to the most recent market offerings, it describes the historical development in research and commercialization for each material. The article examines important properties of several devices, including power diodes, MOSFETs, IGBTs, bandgap, critical electric field, voltage/current ratings, switching frequency, packaging, and dependability. SiC and GaN are given much attention because they compete directly with Si devices due to their bigger bandgaps. Challenges like material availability, thermal management, cost reduction, and gate drive complexity are examined. The current commercial environment is evaluated, following adoption patterns and technological advancements resulting in gains in aerospace, renewable energy, electric vehicles, and other applications needing high-efficiency power conversion [4,5]. The assessment integrates advancements across the wide bandgap power semiconductor spectrum to inform and direct future innovation in this promising field.

## 2. Si

The preceding discussion demonstrates that Si power devices remain the workhorse technology in power electronics applications despite rising competition from WBG power devices. Si is a Group 14 (IVA) member in the periodic table of elements. Si is also part of the carbon family. These family elements include C, Ge, Sn, and Pb. Si is a metalloid, one of only a few elements with metal and nonmetal properties. Aside from oxygen, Si is the second most abundant element on Earth’s crust. Si was established in 1960 by the 11th General Conference on Weights and Measures, CGPM, Conférence Générale des Poids ET Mesures [19]. The CGPM is the international authority that ensures the wide dissemination of Si and modifies it as necessary to reflect the latest advances in science and technology. It has a diamond cubic crystal structure with a lattice parameter of 0.543 nm [20]. The historical overview of Si wafer diameter and crystal weight increase goes beyond the scope of this article. In Table 1, the historical timetable of Si evaluation is presented.

Being a semiconductor, the element, ceramics, and bricks are used for making transistors. It is a vital component of Portland cement. Si materials are used in components of electronic devices. It also makes solar cells [21,22,23,24,25] and parts for computer circuits [26]. A solar cell is a device that converts sunlight into electrical energy [27,28,29,30]. A rectifier is an electrical device that converts alternating current to direct current. The most important Si alloys are those made with Fe, Al, and Cu. When Si is produced, scrap iron and metal are sometimes added to the furnace [31,32]. Several waterproofing systems employ Si as a component for water purification. Si is used in many mold release agents and molding compounds. It is also a component of ferroSi—an alloy widely used in the steel industry.

The diamond cubic crystal structure of Si has a face-centered cubic (FCC) lattice with a basis of two Si atoms. Table 2 overviews Ribbon and Multi-Si technology improvements within the next 5–8 years [33]. However, an analysis of Mono-Si technology was not carried out because the current Mono-Si technology still has too much uncertainty.

### 2.1. Si Diode

For many years, Si diode power semiconductors have been crucial in several applications because of their dependable and effective rectification capabilities. Low forward voltage drops, high current-carrying capacity, and exceptional thermal stability are all positive traits of Si diodes [34]. Their extensive use can be ascribed to their proven dependability records, cost efficiency, and sophisticated production methods. Numerous devices, such as power supplies, inverters, rectifiers, and voltage regulators, use Si diodes. The Si Power Rapid Diode family bridges the gap between SiC diodes. Examples of such Si diodes are previously released emitter-controlled diodes and Infineon’s existing high-power 600 V/650 V diode. Moreover, the TRENCHSTOP™ 5 and high-speed 3 IGBT (Insulated Gate Bipolar Transistor) and CoolMOSTM are good partners for the Rapid 1 and Rapid 2 diodes [35]. For usage in automotive, industrial power control, power management, sensor solutions, and security in Internet of Things applications, Infineon Technologies provides a comprehensive selection of ready-to-use semiconductor design solutions and reference schematics. Si diodes are ultra- and hyper-fast and have outstanding performance with a voltage range of 600–1200 V [36].

As mentioned earlier, the gap between SiC diodes and emitter-controlled diodes is filled by the Rapid 1 and Rapid 2 power Si diodes, which are a complement to the current high-power 600 V/650 V diodes. The new families of hyper- and ultra-fast diodes provide exceptional efficiency and dependability while striking the ideal balance between price and performance. The additional 50 V provides higher reliability.

The 650 V Rapid 1 Diode: The Rapid 1 diode series has the lowest conduction losses, and the smooth recovery minimizes EMI emissions with a 1.35 V temperature-stable forward voltage (FV). The equipment is ideal for power factor correction (PFC) topologies, frequently used in large home appliances like air conditioners and washers.

The 650 V Rapid 2 Diode: The family of Rapid 2 diodes is designed for applications switching between 40 kHz and 100 kHz by providing a low reverse recovery charge (Q_rr_) and time (t_rr_) to reduce the reverse conduction times associated with the power switch turn-on losses and to provide maximum efficiency [37].

### 2.2. Si MOSFET

MOSFETs are extensively utilized power semiconductors that have completely changed the power electronics industry. FETs) have become the most significant device in the semiconductor industry due to Lilienfeld’s 1930 [38] patent on the idea and Kahng and Atalla’s 1960 [39] practical implementation of Si/Si dioxide. The development of this industry has been characterized by an exponential pattern known as Moore’s law over the past seven decades [40]. Today’s metal-oxide-semiconductor field-effect transistor (MOSFET) has undergone several modifications, evolving from a single-gate planar MOSFET to a multiple-gate non-planar MOSFET. Nevertheless, it has been and will continue to be the mainstay of the semiconductor industry for the foreseeable future.

MOSFETs made of Si rely on modulating the conductive channel that forms between a semiconductor layer’s source and drain terminals. The device’s bulk is a Si substrate that has been extensively doped; the gate insulator is a thin Si dioxide layer. A voltage applied to the gate terminal generates an electric field that regulates the channel’s conductivity. A positive voltage repels the majority carriers (for an N-channel MOSFET, electrons) from the channel, resulting in a depletion area and decreasing the conductivity of the channel [41]. Applying a negative voltage draws in most of the carriers and improves the conductivity of the channel. The MOSFET may change between the ON state (conducting) and the OFF state (non-conducting) by varying the gate voltage. This idea makes it possible to effectively manage the MOSFET’s ability to handle power and current flow. These components have low on-resistance and can handle large voltages and currents, making efficient power conversion and control possible [6]. The conductivity of Si MOSFETs may be precisely controlled by using a thin Si dioxide layer as the gate insulator. They offer quick switching times, little gate drive needs, and superior thermal performance.

The main electrical specs for five commercial Si power MOSFETs with current ratings ranging from 2.5 A to 40 A are provided in Table 3. Input capacitance C_iss_ and on-state resistance R_ds(on)_ grow along with the current rating, whereas gate-drain capacitance C_gd_ remains mostly constant. A 2.5 A MOSFET, for example, has a C_iss_ of 1800 pF and R_ds(on)_ of 0.3, whereas a 40 A device has a C_iss_ of 10,000 pF and R_ds(on)_ of 0.05. Across different power MOSFETs, a trade-off exists between a higher current capability and electrical characteristics such as input capacitance and on-resistance. Moreover, this table illustrates how Si power MOSFET specs and performance scale across various current ratings.

Several sectors, including the automobile, renewable energy, industrial automation, and telecommunications, use Si MOSFETs extensively. The subject of power electronics has been profoundly influenced by Si MOSFETs, which are incredibly adaptable and dependable power semiconductors. Si MOSFETs have evolved into crucial components in various applications, from automotive and renewable energy to industrial automation and telecommunications, because of their low on-resistance, high voltage and current handling capacity, and quick switching rates. They are a popular option for power electronic systems due to their superior thermal performance and compatibility with well-known production techniques, ensuring effective power conversion and control. The widespread use of Si MOSFETs demonstrates how important a role they have played in boosting the overall performance and dependability of power electronic systems and devices. They are the favored option for power electronic systems due to their dependability, high efficiency, and compatibility with established production methods.

### 2.3. Si SuperJunction MOSFET

To break the Si 1-D constraint, the super-junction (SJ) concept for vertical power devices was established in the mid-1990s [43,44,45]. When the device is turned off, a vertical P layer or P column is introduced to compensate for the charges in the N drift layer. This approach is highly similar to the RESURF concept [46], which has been used in various lateral power devices. The drift area of these devices has a special design that alternates P- and N-type regions, allowing for a more even dispersion of the electric field. For high-power applications, the SuperJunction design lowers on-resistance and boosts efficiency. SJ MOSFETs are designed to reduce the electric field concentration and provide improved voltage-blocking capabilities by forming a depletion area with several tiny cells [47]. 

Moreover, lower conduction losses, quicker switching times, and enhanced thermal properties are all improved by this design. As a result, the electric field in an SJ device has changed from a triangle to a blue rectangular form, and the N drift layer doping has increased. Forming the vertical P column is the most difficult part of making SJ MOSFET. There are two popular methods, both of which are commercially employed. Si SJ MOSFETs have gained significant attention in power electronics, particularly in applications such as power supplies, LED lighting, and motor drives. Their advanced design and improved efficiency contribute to higher power density and system performance. As a result, Si SJ MOSFETs are rarely used in applications that need a third-quadrant operation, such as voltage source inverters.

### 2.4. Si IGBT

Si IGBTs are critical power semiconductors that have transformed the field of power electronics. These power devices have revolutionized by combining the advantages of MOSFETs and bipolar junction transistors (BJTs) [48]. They offer high voltage and current handling capabilities while maintaining low on-state voltage drop and fast switching speeds. The structure of a Si IGBT comprises three layers: an N-type collector, a P-type base, and an N-type emitter. By regulating the conductivity of the base region through the voltage applied to the gate terminal, the IGBT enables efficient power switching. The performance and efficiency of Si IGBTs have recently been improved for various applications. One key development is integrating cutting-edge trench gate architectures and novel cell designs. These developments have improved switching speeds, decreased on-state losses, and reduced conduction losses. Furthermore, recent research has concentrated on improving the thermal management of the IGBT, enabling better power densities and increased reliability [49]. Due to improvements in power conversion efficiency, increased power density, and system performance, they are now more appropriate for various applications, including electric cars, renewable energy systems, and industrial automation.

Additionally, current Si IGBT advances have concentrated on reaching greater voltage ratings while lowering power losses. One significant development is using cutting-edge gate-driving methods and cell structure optimization in IGBTs. These developments have increased voltage ratings, decreased conduction and switching losses, and enhanced efficiency [50]. Advanced production methods and materials have also made it possible for better thermal management, which has increased power density and enhanced dependability. The latest advancements in Si IGBT technology have opened the door for creating smaller and more effective power electronic systems in various sectors. 

The main features and specifications of popular Si power semiconductor devices, such as BJTs, diodes, MOSFETs, IGBTs, and thyristors, are compared in Table 4. For instance, Si IGBTs have medium conduction, high switching, and overall high-power losses while operating up to 1.2 kV blocking voltage and 50 A current rating. They work well in situations requiring medium voltage and medium frequency. In contrast, despite their slow switching speed, Si thyristors can handle high-voltage, low-frequency applications with up to 4 kV voltage and 3000 A current capacity. Si MOSFETs, which have a blocking voltage of 600 V and a current rating of 100 A, as well as minimal conduction and switching losses, fill the low-voltage, high-frequency market niche. Each Si device has defined features based on its advantages; however, it is constrained by the characteristics.

Table 5 compares four common Si-based power semiconductor devices—diodes, MOSFETs, super junction MOSFETs, and IGBTs—on features like scalability, cost, failure modes, and typical applications, showing the tradeoffs between different devices for use in power electronics and motor drives. MOSFETs and IGBTs are more scalable and suitable for high-power applications like motor drives. At the same time, diodes tend to be lower cost but limited to simpler rectification and voltage clamping circuits.

## 3. Silicon Carbide (SiC)

SiC is a semiconducting material with outstanding physical, chemical, and electrical properties, making it very suitable for fabricating high-power, low-loss semiconductor devices. Moreover, commercially available SiC devices have lower switching/conduction loss, superior thermal stability, and greater temperature tolerance. SiC devices are, therefore, a very promising alternative to converters designed for high-temperature applications [51]. SiC power electronic devices have a theoretically allowed junction temperature of 600 °C due to the semiconductor’s wide bandgap, around three times that of Si material [52]. On the other hand, the fabrication of these devices is rather intricate owing to the same properties of SiC, like its chemical inertness and hardness. It took over a hundred years to develop SiC electronics up to its modern state when power SiC devices possessing higher efficiency than their Si counterparts became commercially available and widely used in numerous applications. The resistance of the SiC material to an electric field is ten times greater than the resistance of the Si. As a result, SiC devices could be considered capable of withstanding the same blocking voltage with a 10-times-thinner material [53].

**Figure 2:**
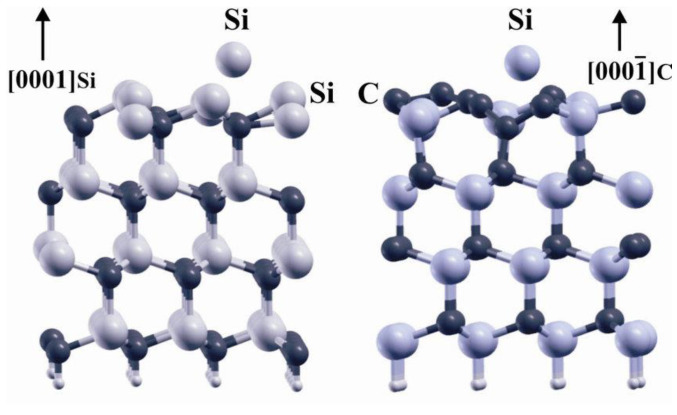
Side view of SiC lattice [54].

A tetrahedral crystalline structure is formed when each Si atom shares its electrons with four carbon atoms. Different SiC poly varieties can be made from this fundamental structure. Shown as Figure 2, SiC is the only chemical compound of group IV elements. It has a strictly stoichiometric concentration ratio of Si and carbon (C) atoms. It should not be mixed with solid solutions, which may be formed by other group IV elements and may have variable component concentration ratios (e.g., SixGe1-x) [54].

SiC devices can operate at higher switching dynamics. The thermal energy required for an electron to pass from the valence band to the conduction band (forming an electron-hole pair) is considerable in WBG materials. As a result, even at high junction temperatures, the WBG device’s electric characteristics are retained within defined bounds. This enables SiC semiconductor devices to function at high temperatures. Currently, the operating temperature range for SiC devices on the market is between 200 °C and 300 °C [55]. Therefore, SiCs are the ideal choice among commercially available WBD devices due to their greater power rating, quicker switching frequency, much lower switching losses, and capacity to handle higher junction temperatures than Si-based devices [54].

### 3.1. Discovery of SiC

As previously mentioned, SiC is an excellent material for high-power electronics and high-temperature applications because it has a wide bandgap and good thermal stability [56]. However, 2D SiC offers incredible new features missing from bulk SiC materials due to quantum confinement and surface effects [57,58,59]. SiC has arguably had the longest history of all the semiconducting materials used in electronics. SiC was found as a manufactured material, which makes its discovery exceptional in and of itself. Jöns Jacob Berzelius (1779–1848), in his 1824 report, most likely made the first observation of a chemical molecule bearing Si–C bonds [60]. He could conduct experiments in peculiar settings because of his acknowledged expertise in experimental methods. He also discovered several new chemical elements, including Si, and many other accomplishments. Berzelius made the extremely cautious claim that he had identified a substance that, when burned, created an equal number of Si and carbon atoms [61].

Seventy years later, the first validated SiC synthesis took place by chance [62]. American engineer Edward Goodrich Acheson (1856–1931) experimented with a newly developed electrical furnace in 1891 to create synthetic diamonds (highly demanded by the industry as an abrasive material) [24]. B. I. Ozernikova and A. P. Bobrievich discovered the first naturally occurring SiC of terrestrial origin in sediments of the Tyung River (Siberia) in 1956 and diamond-bearing kimberlite pipes in Yakutia, USSR, respectively, in 1957 [63]. From Schottky diodes in the 1990s to high voltage SiC MOSFETs and IGBTs in the 2020s, Table 6 traces the evolution of SiC devices, highlighting key developments like the first commercial devices, normally off JFETs, and trench gate MOSFETs that enabled higher voltage, current, and frequency capabilities compared to Si devices for uses like EVs and PV inverters. SiC device advancements and the emergence of GaN/SiC hybrids are boosting adoption and enabling performance above traditional Si power electronics.

### 3.2. Material Growth

Wide bandgap semiconductor SiC has outstanding features that make it highly sought-after for various applications. Epitaxy, the process through which SiC crystals grow, is essential for creating high-performance electronics. Chemical vapor deposition (CVD) and physical vapor transfer (PVT) are two growth techniques that have been used [64]. In CVD, SiC is deposited on a substrate due to the high-temperature decomposition of a precursor gas that contains Si and carbon. SiC source material in PVT is sublimated and recrystallizes onto a colder substrate. To produce high-quality single-crystal SiC, both processes require exact temperature control and a suitable growing environment. Growth rate, shape, and crystal quality are influenced by temperature gradients, gas flow rates, and crystal orientation [65]. Larger, higher-quality SiC crystals with fewer flaws have been made possible by improvements in growth methods and equipment, allowing for the manufacture of power electronic devices, high-frequency devices, and sensors that provide greater performance and efficiency compared to conventional materials [66]. The widespread use of SiC in several technological applications will be facilitated by further research and development efforts in SiC growing processes, which promise future improvements in crystal quality, scalability, and cost-effectiveness.

### 3.3. SiC Diode

Due to the greater SiC dielectric critical field than its Si counterparts, a blocking voltage rise of 10 times above that of Si is achievable with the same thickness of the SiC drift layer. Compared to Si diodes, SiC’s high thermal conductivity has several benefits, including the ability to operate at higher current density ratings and reduce the size of cooling systems. SiC SBDs have been commercially available since 2001 and have continuously increased in the blocking voltage and conduction current ratings. There are essentially three types of SiC power diodes [67]. The PN junction and the Schottky Barrier Diode (SBD) junction are two semiconductor mechanisms that can create a diode, and there is no conductivity modulation in the SBD. From 600 V to 10 kV, Si PN junction diodes dominate the market. The scenario is fully reversed in the case of SBD on WBG material, such as SiC. The high critical electric field E_c_ in SiC reduces the resistance of an SBD significantly. As a result, conductivity modulation is neither necessary nor desirable. Because SBD does not store any charge, it can achieve near-zero reverse recovery loss. As a result, a WBG diode based on the Schottky mechanism is nearly optimal. The junction barrier Schottky (JBS) diode structure, which provides an area to shield the Schottky region in a reverse blocking state, can be used to reduce leakage current.

The PN junction of the JBS diode can become conductive at a high forward bias, giving it a stronger surge capability than the SBD [68]. On the other hand, the SiC PN junction diode will have to overcome a forward voltage drop of roughly 3 V across the PN junction, making it exceedingly undesirable from the standpoint of conduction loss, even if the drift area resistance can be decreased via conductivity modulation. The JBS structure is typically used in SiC diodes above 600 V. Because the off-state leakage current in JBS is decreased, devices can be rated at temperatures as high as 175 °C. Majority of the carrier electrons still are used for device conduction, and advantages of the SiC PIN diode over JBS or SBD is its substantially lower leakage current, which makes it an ideal option for high-voltage and high-temperature operation.

### 3.4. SiC MOSFET

SiC power MOSFETs are quickly gaining widespread application. Reliability issues like bias temperature instability and gate oxide cracking are mostly under control [69]. It is the chosen SiC three-terminal switch due to the well-established gate-driving technique and user base in Si MOSFET and IGBT compared to SiC JFET and BJT. It is commercially available in voltages ranging from 650 V to 1700 V, with higher current (5–600 A) modules [70]. SiC MOSFETs provide clear prospects for improving operating frequency, efficiency, and power density, but their employment is complicated by several unwanted side effects brought on by their rapid switching speed [71]. The characteristics of five commercial SiC MOSFETs are listed in Table 7, along with their component numbers, maximum current ratings in Amps, input capacitances (C_ISS_) in pF, gate-drain capacitances (C_GD_) in pF, and drain-source on-state resistances (R_ds(on)_) in Ohms. SiC MOSFETs can achieve relatively low on-resistances—0.013 Ohms for an 80 A device, which enable high-frequency switching, but at the expense of greater input and gate capacitances than Si MOSFETs.

**Figure 3:**
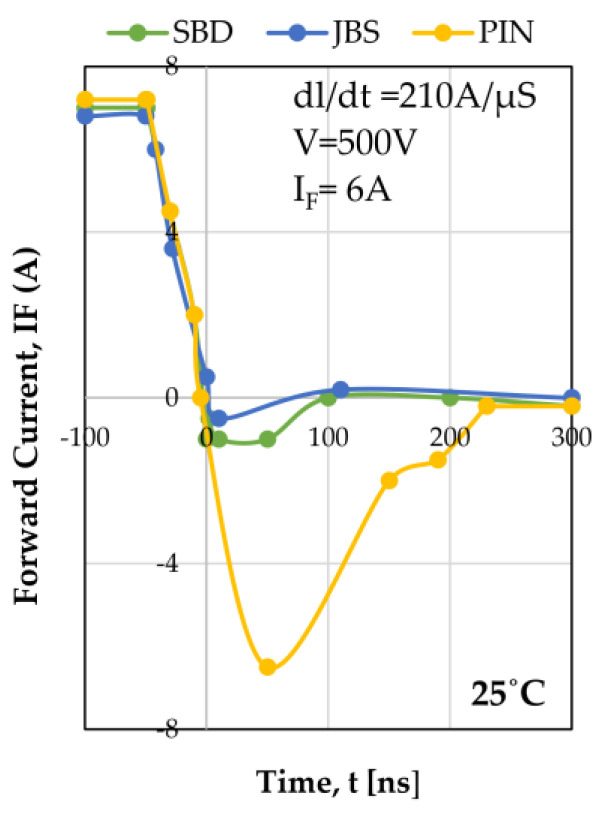
SiC diodes’ turn-off current waveforms at 25 °C inductive load [73].

When compared to the IGBT system, the operation frequency of SiC MOSFET-based converters has increased by one or two orders of magnitude, as demonstrated in Figure 3. SiC MOSFETs can also achieve zero switching loss under specific situations. A 1.2 kV SiC MOSFET module was recently proven to operate at 3.38 MHz [72,73]. The P regions shelter the gate oxide in the planar structure, so the peak electric field near the oxide is decreased. The gate-oxide stability problem in planar MOSFETs has been overcome, and high-reliability performance has been attained [74].

**Figure 4:**
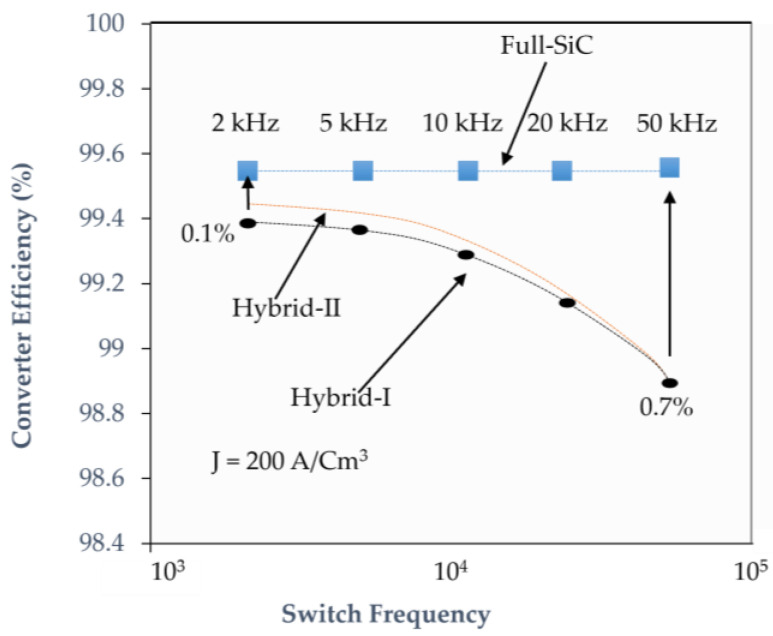
Calculated converter efficiency for a 1200-V SiC MOSFET system versus that based on an IGBT/SiC diode hybrid power module [83].

General Electric (GE) also exhibited the industry’s first dependable SiC MOSFET with a 200 °C junction temperature [75,76]. This is more difficult in the trench device. Many different trench structures exist to protect the trench gate’s bottom [77,78,79,80,81,82]. Another key motivator for SiC MOSFET innovation, in addition to enhancing electrical performance, is reliability. The three key criteria for evaluating dependability are high-temperature gate bias, high-temperature reverse bias, and high-humidity, high-temperature reverse bias. One of the most important challenges in the fabrication of SiC-MOSFETs was the lack of a reliable insulator for the gate terminal. A proper insulator is needed to achieve stable forward I–V characteristics and a stable gate threshold voltage. For this reason, the SiC-MOSFET was commercialized later than the SiC-JFET. Most MOSFETs contain a PIN diode inherent to their structure. This diode has a forward bias voltage around 2.5 V. During the conduction of the body diode, if the MOSFET is turned on, the forward characteristic of the body diode can be virtually improved, and, thus, the conduction losses are reduced. Figure 4 shows the calculated converter efficiency for a 1200-V SiC MOSFET system versus that is based on an IGBT/SiC diode hybrid power module. These graphs are critical for designing power converters for various applications.

Switches, solenoids, encoders, generators, and electric motors are the primary electromechanical devices that connect the digital and physical worlds. The capacity of each of these gadgets to translate electrical impulses into mechanical motions is what gives them their enchantment. The need for more control, efficiency, and capabilities from these electromechanical devices rises as fields like automated manufacturing, electronic vehicles, sophisticated building systems, and smart appliances develop [84]. It investigates how improvements in SiC MOSFETs are expanding the possibilities of electric motors, which hitherto relied on Si IGBTs for power inversion. Similar to the new power electronic converters, SiC devices could be utilized for motor driver applications for novel electric machines for various applications [85,86,87,88,89,90]. This development increases the potential of motor drive applications across all industries.

**Figure 5:**
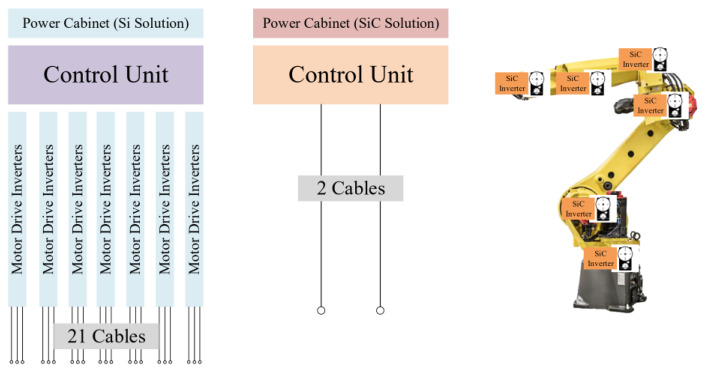
Comparison of a Si IGBT vs. SiC MOSFET system control of a robot arm [84].

The cabling between the drive inverters and the motor driver may be significantly reduced by bringing the motor driver assembly to the motor’s local position, resulting in considerable cost savings. Figure 5 shows seven motors of a robotic arm, that is required to be powered by 21 different cables, which might require hundreds of meters of costly and intricate cabling infrastructure, in a typical Si IGBT power cabinet. Two lengthy cables that link to each motor’s motor drive within the local motor assembly can be used in a SiC MOSFET motor drive system to decrease the number of cables [84]. There are specific applications where IGBTs may still be better suited, as is true for all types of components; however, SiC MOSFET inverters offer several distinct advantages over Si IGBTs, making them very attractive solutions for motor drive and a wide range of other applications.

### 3.5. SiC IGBT

SiC IGBT has received a lot of interest in the domains of high voltage transmission, smart grid, and pulse power since it is the highest voltage switch. SiC IGBTs have not been commercialized because of their inherent flaws and crude manufacturing methods. The stated SiC IGBT devices’ exceptional static and dynamic performance and significant dv/dt during hard switching challenge the power conversion system. SiC IGBT has a lot of potential solutions; however, comparisons with Si IGBT and SiC MOSFET reveal significant discrepancies. The potential SiC IGBT appears to have a chance to displace Si devices in the future based on early experiments in high-voltage fields.

High voltage direct current transmission (HVDC), industrial applications, traction systems, and new pulsed power applications are the key markets for Si IGBT with high voltage and current. Ultra-high voltage in these domains is intended to decrease the number of devices connected in series and simplify converter topologies [91]. According to one source, Si IGBTs can withstand a maximum voltage of 8.4 kV [92], which is about as high as Si devices can go. The continued advancement of Si IGBT in these sectors is also severely constrained by frequency and operating temperature. SiC exhibits greater breakdown field strength, inherent temperature, thermal conductivity, and carrier saturation drift velocity as a wide bandgap material [93]. As can be seen in Table 8, the SiC IGBT device is more competitive in high-voltage, high-temperature, and high-power areas. The SiC IGBT with 4H-polytype and n-channel is chosen among SiC polytypes and channel types because it has a low forward voltage (V_f_), quick switching, and a large safe working region. Wide-base PNP transistors in 4H-SiC N-IGBTs have excellent bulk mobility and low current gain, contributing to a favorable trade-off between V_f_ and switching loss.

SiC IGBTs have a lower V_f_ than Si IGBTs under the same blocking capability but have lower bulk mobility than Si IGBTs. Even though SiC IGBTs have not yet been commercialized, significant advancements have been made over the previous 30 years, as seen in Figure 1. Because of the readily accessible n+ substrate with low resistivity and defect density, the SiC p-channel IGBT has been widely investigated from 1996 when the first SiC IGBT was produced [95] through 2010. Continuous improvements have been made to the P-IGBT’s performance, notably since the charge storage layer (CSL) was introduced [96]. Due to immature technology and a P-type substrate with a high resistivity and defect density during this time, the constructed N-channel IGBT performs poorly [97]. 

Because free-standing technology offers a way to develop a P+ collector on an N+ substrate, the research focus of SiC IGBT shifted to SiC N-channel IGBT before Cooper’s freestanding technology proposal in 2010 [98]. After that, SiC N-IGBT displays ever-improving static and dynamic properties. The SiC IGBT has a blocking voltage that exceeds 27 kV [99], making it a promising device in high-voltage areas. In comparison to the SiC MOSFET of the same rated voltage, the SiC IGBT might cut the differential specific on-resistances (R_on,sp,diff_) by more than one order of magnitude. Therefore, even if the switching loss (E_sw_) of the SiC IGBT is larger than that of SiC MOSFET, it is promising for power conversion systems with more than 100 kW transmission power. Recently, the first solid-state transformer prototypes and the first Marx generators have also been made using the 12–15 kV SiC IGBT modules that were developed [100,101,102].

SiC-IGBTs are more effective than Si-IGBTs because of their switching speed. The gate-to-emitter voltage rise quickly to allow for the SiC-IGBT to switch quickly. Therefore, to charge the input capacitance C_iss_, a greater gate current is needed [103]. Generally, to switch the IGBT off, the gate current capability is increased by reducing the external turn-on and turn-off gate resistors even though the same current capability is needed. In order for the gate current to rise as quickly as required, the gate driver’s stray inductance must also be kept to a minimum [94]. SiC-IGBTs need a negative gate-to-emitter voltage like that found in Si-IGBTs to obtain a quick and secure turn-off transient. A SiC-IGBT driver typically supplies a gate-to-emitter voltage of +20 V positive and 5 V negative [104].

SiC BJTs, JFETs, Schottky diodes, and MOSFETs are four popular SiC power semiconductor devices described and shown in Table 9. SiC devices outperform Si in high-voltage, high-frequency applications like PV inverters, EV systems, and power supplies. It compares parameters like bandgap, electron mobility, voltage/current ratings, switching frequency, power losses, and figures of merit. The table shows the compromises made by various SiC devices for application-specific performance optimization.

### 3.6. Applications and Emergence of SiC Power Electronics

SiC is employed in semiconducting and other items such as armored vehicles, ceramic plates, thin filament pyrometry, foundry crucibles, and auto clutches. SiC was initially used in electrical applications as a lightning arrester in a high-voltage power system because engineers and scientists realized SiC works well even in the presence of high volts and high temperatures. SiC devices are appropriate for a wide range of applications in aerospace and space missions [105,106], despite the necessity for high-temperature dependable device packaging to be developed [107,108]. Schottky diodes, MOSFETs, and power electronics are some of the most recent electrical devices that use SiC.

Applications for SiC include sandblasting injectors, automobile water pump seals, bearings, pump parts, and extrusion dies. These applications use SiC’s exceptional hardness, abrasion resistance, and corrosion resistance [109]. SiC is undoubtedly durable and versatile, with applications ranging from semiconductors for Schottky diodes to use as an abrasive polishing material. Its exceptional qualities include sublimation, great chemical inertness and corrosion resistance, excellent thermal characteristics, and the capacity to develop as a single-crystal structure.

The introduction of the first mass-produced electrical vehicles (EVs) to the market in 2008—Tesla Motors’ debut of its first all-electric vehicle—significantly impacted the development of SiC power electronics. Two components of the electrical power train significantly impact the performance of these cars. They are an inverter and a battery charger that transform the DC power from a battery pack into AC power for a motor. Since the battery capacitance in EVs restricts the amount of on-board stored energy, the efficiency of power conversion by these units is crucial. The bulk of modern electric vehicles (EVs) and the first electric vehicles (EVs) used inverters with Si IGBTs and conversion efficiencies ranging from 80% to 95%. Even at 95% efficiency, these inverters waste too much energy and need liquid cooling. Compared to the electrical motors, they are bigger and heavier. The conversion efficiency of the inverter might be increased to 99% [110] by swapping Si IGBTs for SiC MOSFETs while being significantly lighter and smaller. The first commercial SiC power MOSFET was introduced by Cree, Inc. in 2011 [111]. This potential use of SiC power devices in the high-volume automotive sector sparked increased research into the design and technology of SiC devices.

Tesla introduced the Model 3 in 2017, the first electric vehicle to have inverters based on SiC MOSFETs. By the time this article was published, each week’s manufacturing of automobiles used 48 SiC MOSFETs with a 650 V/100 A rating made at the STMicroelectronics fab in Catania, Italy [112]. At the same time, Tesla Model 3’s traction motor spins at 17,900 revolutions per minute, and China’s NEV Technology Roadmap 2.0 aims to reach a motor speed of 25,000 rotations per minute by 2035 [113]. DENSO, a global automotive manufacturer, built its first inverter using SiC semiconductors. The new Lexus RZ, a Toyota luxury brand’s first specifically designed battery electric vehicle (BEV) model, will employ this inverter, which is part of the eAxle, an electric drive module designed by BluE Nexus Corporation [114]. It appears that Toyota is producing its first BEV in large quantities through the Lexus Division. SiC and GaN are competing to see which is superior for power electronics. They both outperform Si in either situation.

The market for SiC power devices is expanding rapidly, and the SiC industrial sector today exhibits significant diversity, with successful businesses operating in various ways. This predicts that SiC power electronics will continue to flourish as an industrial technology during the coming several decades. The enormous potential of SiC as a material for high-temperature and high-frequency electronics, which is still not realized and is awaiting convincing demonstration of SiC’s superiority over conventional semiconductors for these applications, is another factor driving the further development of SiC technology. 

Over the past 13 years, GE Aviation has committed over $150 million to developing SiC technology, solidifying its position as the market leader. Because of characteristics like high-temperature tolerance, low losses, and higher frequencies, SiC enables lighter, more effective, and higher-performance power electronics. To reach extremely high-power density and efficiency goals, GE is utilizing SiC technology in aerospace applications, including hybrid electric aircraft propulsion. GE can create lighter and more potent systems for electric ground vehicles and other applications due to SiC. GE Aviation is well-positioned to promote SiC power device adoption moving ahead due to its extensive aerospace knowledge and experience.

The SiC-based generator controller developed by GE Aerospace shows a significant advancement in power electronics when compared to industrial Si-based generator controllers, as shown in Table 10. Its benefits are glaringly obvious in many important areas. First off, the SiC controller is extraordinarily effective at harvesting and managing electrical power within a small footprint due to an amazing fourfold improvement in power density relative to its size. With a twofold increase in power density per unit weight, its power-to-weight ratio is also improved, which results in both increased efficiency and a decrease in the overall weight of the controller. This drop is accompanied by a 50% reduction in physical size, highlighting SiC’s promise for applications that conserve space. Notably, the SiC-based controller excels at increasing conversion efficiency. It increases DC/AC conversion efficiency from 94% to an astounding 99%, guaranteeing little energy is lost in the process. Similarly, it improves efficiency in DC/DC conversions from 84% to 95%, maximizing power transfer. SiC technology improves efficiency in AC/DC conversions from 85% to 92%, leading to more effective power conversion and energy use.

**Figure 6:**
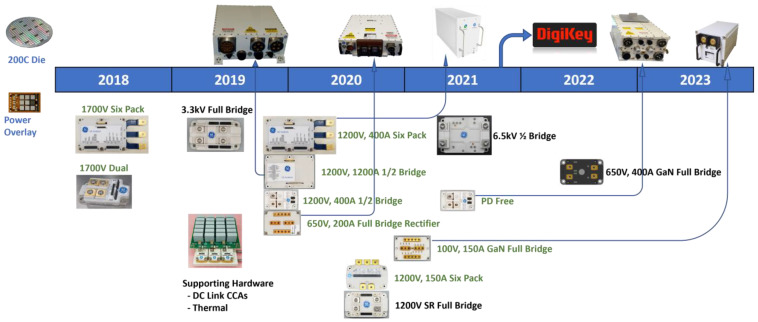
Evaluation of wideband power semiconductor modules designed and manufactured by GE Aerospace [115,116,117].

Figure 6 shows the evaluation of SiC modules and SiC technology utilized in aerospace applications by GE Aerospace in conjunction with Power America, the Department of Energy, and the National Renewable Energy Laboratory. Specifications for various SiC power module variants from GE Aerospace are listed in Table 11. The modules have voltage ratings of 1200 V and 1700 V and come in half-bridge, dual-bridge, six-switch, and six-pack versions. Ratings range from 425 A to 1425 A. On-state resistance, thermal resistance, size, and maximum junction temperature are important factors mentioned. With small footprints up to 100 mm × 140 mm and operational temperatures up to 175 °C, the table demonstrates great power density and temperature capability of SiC modules. SiC modules can be configured in a wide range of ways to accommodate various power electronic circuit topologies and the current/voltage ratings needed for aviation applications.

### 3.7. Challenges of SiC Power Device Development

Although high voltage (HV) SiC devices perform better than their Si counterparts, additional considerations must be made to use them effectively. The HV SiC device application must overcome obstacles in a variety of time frames. The majority of difficulties are caused by relatively brief time intervals (between 0.01 and 1 μs), such as electromagnetic interference (EMI), packaging, and gate drive design. The output of a SiC-based converter can have advantages in terms of output with a high-frequency range, low total harmonic distortion (THD), and high control bandwidth, to name a few. Newly developed converter topologies and PWM schemes could be utilized with SiC devices to compare the viability of the device [118,119,120,121]. HV SiC device application technology is still in the early stages of development [122]. The material’s properties present a significant problem for SiC manufacturing. Generally, SiC takes more energy, longer, and higher temperatures for crystal growth and processing due to its extreme hardness, which is nearly diamond-like [123].

#### 3.7.1. Packaging

**Figure 7:**
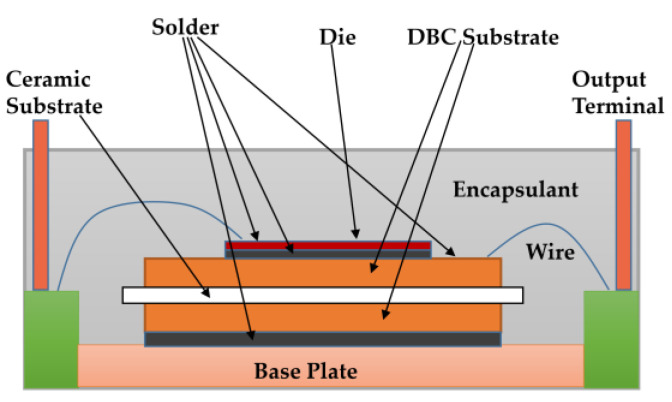
SiC power module packaging structure.

Building power modules with small and fast device dies requires addressing electrical, thermal, and mechanical challenges. Figure 7 depicts a typical power module packing.

#### 3.7.2. Electrical Insulation

The packaging of HV SiC power modules presents a significant problem regarding electrical insulation design. Special attention should be given to vulnerable places that are easily compromised, such as the die, substrate, and output terminal. SiC devices have substantially greater voltage ratings than Si devices, although SiC devices have thinner dies. The electric field around the die from the anode to the trace that is soldered with the cathode gets noticeably stronger with the higher voltage rating and thinner die. A recent generation 10 kV SiC MOSFET, for instance, has a die thickness of 100 μm and an average electric field of 100 kV/mm [124], but a 1.7 kV IGBT has a die thickness of 209 μm and an electric field of only 8.1 kV/mm [123]. As a result, HV SiC device packaging surrounding the die has an electrical field concentration that is ten times greater than Si’s.

#### 3.7.3. Parasitics

The internal connecting wires inside the module and the external power stage contain the stray inductance. The combination of these stray inductance components affects how well the gadget switches. Using a decoupling capacitor [122,123] can minimize the influence of stray inductance in the external power stage. The stray inductance inside the module is the main subject of this subsection. They primarily have three effects on device performance: voltage overshoot, ringing, and switching speed restriction.

#### 3.7.4. Power Module with Multi-Dies

Developing the multi-die in parallel connection for HV SiC-based power modules is important due to the single die’s restricted current rating of present HV SiC devices, typically approximately 20 A per die. For instance, the 10 kV/240 A SiC MOSFET power module is made up of 18 dies connected in parallel [125].

#### 3.7.5. Gate Drive

The gate drive connects the power semiconductor device and control. Power semiconductors are turned on and off by transferring the control signal from the gate drive to the drive signal. Gate drive design is crucial for SiC devices to achieve their maximum potential. Two crucial factors, efficiency and dependability, should be considered when designing the gate drive. The HV SiC devices do not have commercially accessible gate drivers because they are unique power devices. Its gate drive needs to be the subject of research [122].

#### 3.7.6. Electromagnetic Interference

The converters using HV SiC devices may experience significant EMI due to the high dv/dt caused by the quick switching speed and the huge parasitic capacitance due to the compact size. Both EMI filters and special EMI reduction techniques are desirable. The EMI and dv/dt filters in motor drives are essential for preventing the voltage doubling effect that results from high dv/dt [126]. High dv/dt will significantly increase the grid-side conduction EMI in applications involving the grid. EMI filters are required to ensure that the power conversion system satisfies the criteria for grid-connected converters [127].

### 3.8. Future Trends of SiC

SiC devices have several advantages and many difficulties from both the device and application viewpoints. These serve as a guide for upcoming developments in research:

#### 3.8.1. Voltage-Derating Guidelines

A voltage-derating design guideline must be devised and SiC devices’ field dependability for various applications must be shown. This is particularly crucial for applications like aviation systems [127], where reliability is essential. On the one hand, a SiC device in a power conversion system may have a greater current rating than a Si device, resulting in a larger thermal ripple. On the other hand, the SiC device’s ability to operate at greater temperatures suggests stricter requirements for the packaging materials.

#### 3.8.2. Improving Manufacturing Techniques for Affordability

To make SiC devices more affordable for system applications, manufacturing techniques must be improved for higher yields [128]. The majority of significant SiC producers are switching to 150 mm SiC epitaxial wafers. There are also debates over switching to a fabless manufacturing method, which would allow SiC power devices to be produced in Si fabs. Because the quality would be ensured by the already existing facilities and established production infrastructure, such a shift would result in lower prices. It is difficult, nevertheless, due to the rigidity of SiC equipment and processes.

#### 3.8.3. Considerations for SiC Device Gate Driver Design

SiC devices switch significantly faster than Si devices, which presents difficulties for the design of the gate driver [129,130]. First, the SiC device’s larger dv/dt injects more common mode current through a miller capacitor into the gate loop, creating a positive spurious gate voltage. An improved gate driver with active dv/dt and di/dt control is a growing trend. Additionally, a greater dv/dt injects a higher common mode current through the isolation barrier to the primary side of the gate driver, limiting the coupling capacitance. Second, SiC devices have a smaller die size and a faster current rise under the fault. Thus, the gate driver’s short circuit protection response requirement is larger, and the SiC device’s parallel or series connection is more susceptible to timing errors.

#### 3.8.4. Novel EMI Filter Design

It is necessary to look at the novel EMI filter design [108]. The EMI noise is 10 to 100 times higher due to the faster switching transient and higher switching frequency. When used in high-voltage and high-power applications, this becomes very difficult. Firstly, SiC power converters require the modeling and prediction methods of EMI noise. On the other side, novel filter and shielding designs are required.

#### 3.8.5. Reducing Commutation Loop for Enhanced SiC Device Performance

Using novel system designs with a reduced commutation loop is crucial, such as the SiC device package and system busbar structure [72]. Although it is known that a SiC power converter may supply more current, this is only true when the breakdown voltage of the device is lower than the device voltage stress during the transition. In other words, the voltage overshoot may restrict the system’s current rating rather than the thermal. This issue is extremely significant for some new applications, such as the 1.5 kV DC photovoltaic system [131]. Up until now, laminated and multi-layer laminated busbar structures have been the preferred option. Investigating a low-inductance capacitor is also necessary.

#### 3.8.6. Exploring Cooling Techniques for SiC Device Reliability

SiC chips’ lower die size and, thus, increased loss density present new design difficulties for heat management techniques [132]. Additionally, the SiC chips’ lower thermal capacitance could lead to a larger temperature ripple, which could present problems from a reliability standpoint. Phase change cooling, liquid jet impingement cooling, and other effective cooling techniques are some prospective, promising alternatives [133]. The advancing 3D printing technologies may also be leveraged to support original ideas.

#### 3.8.7. Advancements in Ancillary Components for High Temperature

SiC power devices’ capacity to operate at high temperatures necessitates advancements in ancillary components such as high-temperature capacitors, packaging, control electronics, gate drivers, and sensors [134]. The bus bars that connect the high-temperature devices to the capacitors make them particularly difficult since they raise the temperature of the capacitors even further. To make the capacitors usable with high-temperature switches, cooling may be required. In addition, Si-on-insulator (SOI) or SiC-based high-temperature gate drivers are required. The typical temperature range for SOI technology is 225 °C or lower. Since SiC has a lower channel mobility than other semiconductors and is, therefore, not appropriate for very low-voltage applications, SiC-based integrated circuit design is complex.

#### 3.8.8. Lowering Engineering Effort and Costs

Due to their lack of familiarity with these novel devices, many potential SiC users are also concerned about the expensive non-recurring engineering expense. The conventional SiC PEBB, like the Si PEBB, can dramatically lower engineering effort and development costs, opening the door for commercializing SiC applications [135]. The heat sink, the robust gate driver, the high-bandwidth and noise-free controller, and the low-inductance busbar may all be incorporated. The entire system might then be constructed entirely around this PEBB for various purposes.

## 4. GaN

Due to its capacity to provide much better performance across a variety of applications while using less energy and physical space to do so compared to current Si technologies, GaN is gaining significance. GaN technologies are becoming crucial in some applications where Si as a power conversion platform has reached its physical limits, while in other applications the advantages of efficiency, switching speed, compactness, and high-temperature operation combine to make GaN increasingly alluring. 

Table 12 depicts the evolution of GaN power semiconductor devices from 1990s HEMTs to current MISFETs (Metal Insulator Semiconductor Field Effect Transistor) and IGBTs, highlighting significant developments such as the introduction of the first enhancement mode transistors, the dependability of the GaN MISFET gate oxide, and the extension into high-voltage EV applications. With growing vertical GaN, MISFETs, and GaN-on-Si, which overcome the limits of early HEMTs for better voltage ratings and reliability to replace and exceed Si MOSFETs and IGBTs, GaN enables higher-frequency switching and current density compared to Si devices.

GaN-based power devices first appeared in 2000, with the GaN FET being manufactured on a SiC substrate utilizing radio frequency standards. Following that, with the advancement of material growth techniques, GaN power devices have made a quantum jump in quality [136]. GaN has a strong atomic junction because it is made up of nitrogen, a light element. Their net parameter, which refers to the distance between atomic unit cells and their crystalline structure within the same material, is less than that of other semiconductors from the III–V groups of the periodic table [137]. As a result, GaN has better electrical properties than Si dioxide.

**Figure 8:**
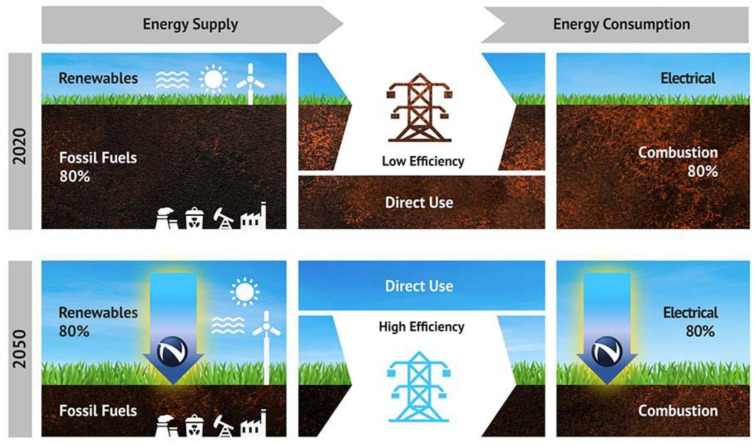
GaN technology to significantly reduce carbon emissions and energy consumption [138].

A switch to GaN technology will assist in fulfilling demand while reducing carbon emissions as the world’s energy needs grow. It has been demonstrated that the design and integration of GaN may provide next-generation power semiconductors with a carbon footprint 10 times less than that of slower, older Si chips, as shown in Figure 8. In order to strengthen the argument for GaN, it is predicted that switching all data centers from Si to GaN will cut energy loss by 30–40%, resulting in savings of over 100 TWhr and 125 Mtons of CO_2_ by 2030 [138].

GaN has appeal for reasons other than only operational performance and system-level efficiency gains. GaN offers a strong “green” edge over older, slower Si because a GaN power IC chip could save 80% in manufacturing and process chemicals and energy and more than 50% in packaging.

Currently, GaN wafer diameters of 2″ have been obtained at a cost that is 10 times that of SiC and up to 100 times that of Si [139]. However, in contrast to SiC, GaN has been widely used in optoelectronics and radio frequency applications due to its broad energy band and potential high-frequency properties [140]. A lot of electronics, such as radio transmitters, plasma generators, MRI scanners, power converters, and wireless power transfer (WPT), among many others, depend on radio frequency (RF) power [141]. GaN’s wide bandgap, huge critical electric field, strong electron mobility, and reasonably good thermal conductivity make it appealing for high-voltage, high-frequency, and high-temperature applications. GaN-based materials can emit light in various visible wavelengths (violet, blue, and green), as well as for high frequency and high-power applications, due to their wide range of energy bandgap [142,143,144].

GaN and its alloys, such as AlGaN and InGaN, were the subject of much research. Other options are used, including Si, sapphire, and SiC substrates, which are less expensive. These substrate materials match the lattice properly and are thermally compatible with GaN. GaN-based devices are currently commercialized in the photonics field, although this semiconductor material is still in the early stages of development for power applications. Due to their cheaper cost, GaN rectifiers on Si or sapphire substrates, and their remarkable trade-off between on-resistance and breakdown voltage, these devices have gained much attention in recent years. For 600 V and lower voltage applications, the GaN heterojunction field-effect transistor (HFET) has been commercially introduced [145,146]. The source and drain contacts are interconnected using many metal layers. Another notable WBG material is GaN, which has features ideal for power device applications. Early GaN HFETs are depletion mode devices; to achieve an enhancement device, a cascade setup with a Si MOSFET will be required [146].

Over the past ten years, GaN has risen to the top of the materials used to make power devices. Next-generation power devices use the compound semiconductor material GaN. Due to its advantages over Si-based devices, such as outstanding high-frequency characteristics, it is starting to be adopted [147]. GaN is a fantastic material for fabricating high-speed/high-voltage components since it has the widest energy gap, critical field, and saturation velocity among semiconductors for which power devices are already on the market [148]. Without doping, a two-dimensional electron plasma with high mobility and a high channel density is created using AlGaN/GaN heterostructures. The presence of spontaneous and piezoelectric polarization makes this possible.

Additionally, the ability to build these devices on large-size, cheaper Si substrates offers a financial benefit that enables one to use Si CMOS equipment and affordable facilities [149]. Subsequently, due to their low switching charges and parasitic capacitances, GaN transistors have lower switching and resistive losses [148]. Device scaling and monolithic integration, which have advantages in terms of downsizing, enable a high-frequency operation. Since GaN is a more recent material than Si, it is crucial to fully understand and characterize the trapping and deteriorating processes in order to increase device stability and dependability [148]. GaN-based devices are currently commercialized in the photonics field, although this semiconductor material is still in the early stages of development for power applications.

### 4.1. GaN Diode

The advancement of the GaN substrate in recent years has been the driving force behind the development of vertical GaN-based devices. The vertical GaN diode, on the other hand, has, at this early stage, become a hot research area due to the relatively immature technology for the vertical triode [150]. Vertical GaN SBDs are similar to AlGaN/GaN SBDs in that they both exhibit low conduction loss and high switching speeds with little reverse recovery time in frequency fields [151,152]. However, the latter has a higher current density and fewer leakage paths.

The bulk of GaN Schottky power diodes disclosed up to this point feature either lateral or quasi-vertical layouts because there are not many GaN substrates with electrical conductivity [153]. Although the forward voltage drop is still significant, lateral GaN rectifiers have shown breakdown voltages as high as 9.7 kV on sapphire substrates [154]. GaN rectifiers constructed on Si or sapphire substrates are quite popular because of their lower cost. Due to the recent availability of high-temperature HVPE (hydride vapor phase epitaxy) free-standing GaN substrates, 600 V operating voltage GaN Schottky diodes are soon to be made available on the market to compete with SiC Schottky rectifiers [155]. In addition, commercial GaN Schottky diodes operating in the 600 V to 1.2 kV voltage range will be made available by the industry very soon. JBS GaN diodes, however, could enhance the performance of GaN-based power rectifiers in the 600 V to 3.3 kV range; however, contact resistance to implanted p-type GaN still needs to be reduced [156].

There are two types of GaN power diodes: the GaN Schottky barrier diode (SBD) and the GaN power diode (PN). When a typical GaN power rectifier is turned on, electrons must pass over the Schottky barrier of the SBD, resulting in a high turn-on voltage that is incompatible with lowering device losses [151]. Rapid progress in the growth and fabrication of vertical GaN (v-GaN) diodes has been made in the past few years, with the device unipolar figures of merit (UFOMs) exceeding those of SiC [157,158,159,160]. As the peak electric field is present primarily in the bulk rather than along the surface as in lateral devices, v-GaN diodes can operate in systems requiring higher-voltage hold-off. This enables competition with SiC and Si diodes in voltage regimes above 600 V [161].

The vertical structure is extensively employed in general-purpose power electronic devices that can provide a higher current. This structure includes the benefits of both lateral and vertical structures, but it also has the downsides of both. Its advantage is that it can be used with existing processes and can be created in large sizes [162]. Dang et al. reported Au/Pt-GaN Schottky diodes with up to 550 V breakdown voltage in 2000 [163]. In 2009, Arslan et al. used the metal-organic chemical vapor deposition (MOCVD) technology to make a Ni/Au-AlGaN/GaN heterojunction Schottky diode and examined its current transport under various temperature settings [164]. The first GaN commercial integrated power devices, the iP2010 and iP2011, were developed in 2010 by US International Rectifier, based on the GaN SBD technology platform—GaNpowIR. Micro GaN, a German business, launched the 600 V line of products for high-power, high-voltage applications in 2010, which included the Schottky diode MGG1TO617. Its turn-on voltage, turn-on resistance, and drain-source voltages are all less than 0.3 V, 329 m, and 600 V, respectively, and the leakage current is only 1 mA, reducing switching losses significantly. It has been used in aerospace and defense, power conversion, and traction application [165].

In 2011, EPC Corporation released its GaN line of products, with a maximum voltage of 300 V and a low on-resistance of only 150 m. Sanken Electric, a Japanese company, uses GaN-based SBD and HEMT solutions in DC/DC converters and plans to introduce 600 V diodes in 2012. Panasonic and Sharp have since released 600 V GaN-based SBDs. With the help of ARPA-E (American Energy Advanced Research Projects Agency) and the military, Avogy, now Nexgen Power Systems, is rapidly expanding its product line to include not only 600 V GaN SBD commercial products but also 1700 V PN-type diodes, which are used in solar and wind energy inverters, electric vehicles, power conversion, and aerospace applications [166]. Avogy’s commercial GaN diode products are shown in Table 13. Because the technique for forming PN junctions on GaN materials is still in its beginning, in order to improve device performance and get over the limits of basic GaN SBDs and p-n diodes, several high-tech vertical GaN power rectifiers were created. Basic p-n diodes have a high forward voltage drop and a big reverse recovery current, whereas basic SBDs have significant reverse leakage and low breakdown voltage as downsides [167].

### 4.2. GaN MOSFET

Five commercial GaN MOSFETs’ characteristics are included in Table 14, along with their component numbers, maximum current ratings in Amps, input capacitances (C_ISS_) in pF, gate-drain capacitances (C_GD_) in pF, and drain-source on-state resistances (R_ds(on)_) in Ohms. The high electron mobility and current density of GaN enable GaN MOSFETs to achieve extremely low on-resistances, such as 0.035 Ohms for a 40 A device, as shown in the table. However, compared to the equivalent Si MOSFETs, input and gate capacitances are larger, which can reduce the performance of high-frequency switching.

For the vertical GaN MOSFETs, the cell pitch of our GaN MOSFETs is around 3–5 times larger than that of the SiC MOSFETs [168]. At the same time, lateral GaN MOSFETs benefit from consistent and broad conduction band migration in high-voltage power switching, making them less vulnerable to hot electron injection and a better replacement for SiC MOSFETs and GaN HEMTs. Although our GaN MOSFETs’ cell pitch is too wide, the performance of the vertical GaN trench MOSFETs is in line with the top SiC MOSFET performance. However, compared to SiC devices, the doping concentrations of GaN FETs are relatively lower [169].

### 4.3. GaN Heterojunction Field-Effect Transistor (HEFT)

GaN heterostructure field-effect transistors (HFETs), a kind of wide bandgap semiconductor electronic components, are popular in high-frequency and high-power applications due to their advantages of having a high breakdown voltage and high electron mobility [134,135]. Vertical power devices based on GaN material are still at a very early research stage [136,137,138,139,140,141,142,143,144,145,146,147,148], and there are currently no commercial vertical power devices accessible due to the difficulty in producing low-cost GaN epitaxial wafers, which is required to construct vertical power devices.

The market adoption of normally off GaN-based power electronics solutions will also be influenced by the reduction of material costs and the enhancement of material quality, both of which impact the dependability of the devices. Accordingly, significant research efforts by the scientific community would be needed over the following years to achieve a thorough knowledge of the physics of GaN-based materials and devices [149]. Nitride-based electrical devices show great promise due to their high electron mobility and saturation velocity, high sheet carrier concentration at heterojunction interfaces, strong breakdown field, and low thermal impedance of GaN-based films produced over SiC or bulk aluminum nitride (AlN) substrates [150]. It is predicted that the specific on-state resistance (R_on_) of FETs will be lower than that of Si or gallium arsenide. FETs with very low on-state resistance are very effective for low-loss power-switching devices like inverters.

### 4.4. GaN High Electron Mobility Transistor (HEMT)

GaN HEMTs are aggressively used in high-performance compact power supplies for fast chargers, data centers, light detection and ranging (LiDAR), and other applications because they exhibit exceptional performance as power-switching devices, including ultrahigh switching frequencies and high conversion efficiency [16,170]. It is important to note that the on-state losses and switching losses can greatly decrease while maintaining the desired normally off feature in a cascade GaN HEMT built from a high-voltage D-mode GaN HEMT and a high-speed, low-voltage, and Si MOSFET [170]. It is rather relatively simple since controlling a cascade GaN HEMT is identical to driving a Si-MOSFET. On the other hand, due to E-mode GaN HEMT’s high voltage and current slew rates, low threshold voltage, and low allowed gate voltages, operating an E-mode GaN HEMT requires considering several complex parameters. A negative driving voltage can be utilized to assure successful turn-off operations, although it may increase reverse conduction loss and require additional power supply units. A decent alternative is a voltage clamp [170].

Due to their rapid switching speed and low conduction losses, GaN HEMT are particularly appealing for high-frequency applications. Because there are no impurities (dopants) in the 2DEG area, electron mobility is high, allowing for low resistance and quick switching. The majority of GaN-HEMT transistors are lateral structures. In the heterojunction formed by GaN, the polarization electric field significantly modulates the distribution of energy bands and charges; GaN heterojunction field effect transistors dominate the GaN transistor. The device structure is also called HEMT [171].

#### 4.4.1. Enhanced GaN HEMT

Due to the polarization characteristics of the conventional GaN HEMT, in the most used voltage-type power converter, power switches are required to be in a normally off state from the perspective of safety and energy saving, so a lot of research work is now focused on implementing enhanced GaN HEMT devices. When the enhanced GaN HEMT is in a normally off state, short circuit elimination techniques are widely used for protection [172,173,174]. At present, large international semiconductor companies, such as America’s MicroGaN, Transphorm, EPC, Germany’s Infineon, Japan’s Panasonic, and Canada’s GaN systems, have introduced GaN HEMT devices, the highest voltage reaching 1200 V.

#### 4.4.2. High-Voltage Cascade GaN HEMT

**Figure 9:**
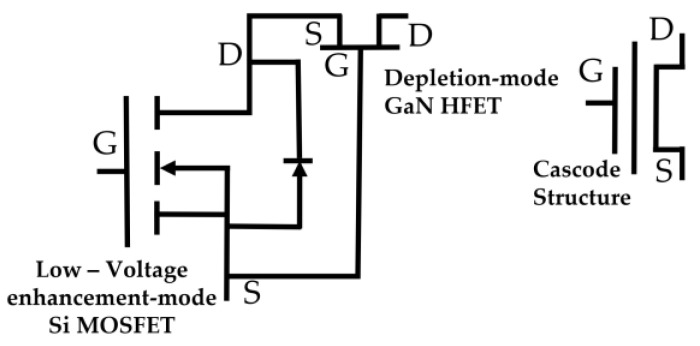
Device structure of cascade GaN HEMT.

As mentioned, GaN devices now play a role in high-voltage applications due to the introduction of high-voltage cascade GaN HEMTs. As illustrated in Figure 9, the cascade structure combines a high-voltage normally-on GaN HFET and a low-voltage normally-off type Si MOSFET into a new mixing tube, resulting in a normally off state [175]. It is a voltage-controlled device in which the gate is activated, 2DEG is produced, and the transistor is turned on when the negative voltage between the gate and the source is greater than the threshold voltage. The transistor switches off when the voltage between the gate and the source is less than the threshold voltage. Because the on-state and switching losses of Cascode GaN HEMTs are relatively low, and the diode has stronger reverse recovery properties than Si MOSFETs, the power system’s efficiency can be greatly improved [175].

Table 15 lists the parameters for GaN HEMTs, GaN MISFETs, and GaN Schottky diodes. Bandgap, electron mobility, voltage/current ratings, switching frequency, and power losses are just a few comparisons made. High critical electric field, high current density, and high frequency switching are some of GaN devices’ key benefits over Si, while thermal conductivity and lattice mismatch still present problems. GaN HEMTs and diodes enable high-frequency performance, and MISFETs, which aim to replace Si MOSFETs and IGBTs, offer greater dependability.

### 4.5. Applications of GaN

Long-employed in the manufacture of RF and LED components, GaN is now becoming more widely accepted in various power-switching and conversion applications. GaN-based ICs may meet this requirement by delivering reliable operation at greater temperatures, saving space, and enhancing system performance and efficiency. The innovative semiconductor material GaN has received much attention and is used extensively in many different sectors. GaN outperforms conventional Si-based devices in terms of power efficiency, speed, and durability due to its special characteristics, including high electron mobility, broad bandgap, and high breakdown voltage.

**Figure 10:**
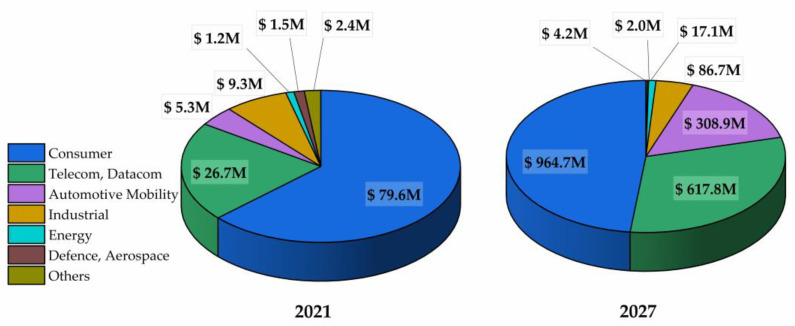
2021–2027 power GaN device market projected revenue [176].

The power GaN development market is predicted to increase from $126 million in 2021 to $2 billion in 2027, at a compound annual growth rate (CAGR) of 59%, according to market research firm Yole Développement [176], which is shown in Figure 10. The consumer sector, which includes fast chargers, Class-D audio, power banks, and time-of-flight sensors in smartphones and tablets, will contribute significantly to this expansion. GaN offers greater power density, improved thermal efficiency, and more compact and lightweight solutions in various applications. The convergence is driving growth in the telecom/datacom and automotive/mobility sectors to a 48 V power supply in both high-power density computers and vehicle DC–DC converters. Data centers need less power when using 48 V systems, and GaN performs better under these conditions than Si does. Manufacturers of solar microinverters, optimizers, and energy storage systems are increasingly developing using GaN for improved efficiency, higher power density, and increased dependability as the adoption of renewable energy sources quickens. A number of firms, including BRC Solar and Solarnative, have introduced GaN-based solutions.

There is a critical need for energy-efficient solutions due to the expansion of the cloud and rising demand for data centers. Using GaN technology, which enables a single-stage power conversion process and eliminates the intermediary step of converting power from 48 V to 12 V, is one option to deal with the problem. Significant energy savings are provided by this direct conversion from 48 V to the necessary 1 V at the point of load. GaN technology is also used in autonomous cars, especially in LIDAR systems. GaN technology accelerates the transmission of laser beams, improving lidar’s resolution and mapping capabilities and paving the way for autonomous vehicles and augmented reality applications. Devices made of GaN are widely used in a variety of industries. GaN is useful for ion thrusters, solar power conversion, robotics, and lidar in space due to its built-in radiation tolerance. GaN is also transforming motor drives, making it possible for eMobility, personal robots, and drones to have smaller, lighter, and more effective systems [176]. GaN-based power solutions improve the efficiency and dependability of solar micro-inverters and energy storage devices in renewable energy. GaN’s wireless power sources aid medical technology by enhancing implanted device charging and allowing portable imaging equipment for procedures like colonoscopies and MRI scans. The development of wireless power, which enables wireless device charging and is revolutionizing our way of life for everything from smartphones to home appliances, is also being fueled by GaN.

GaN RF components are used in phones and laptops to send and receive GSM and WiFi signals, and GaN is increasingly being used in the chargers and adapters that power these devices. The mobile fast-charging industry is the biggest market for power GaN at the moment. GaN power ICs can enable three times quicker charging in adapters that are half as big and heavy as sluggish, Si-based solutions. Additionally, the retail launch price of GaN for single-output chargers is around half that of earlier best-in-class Si chargers and up to three times cheaper for multi-output chargers.

**Figure 11:**
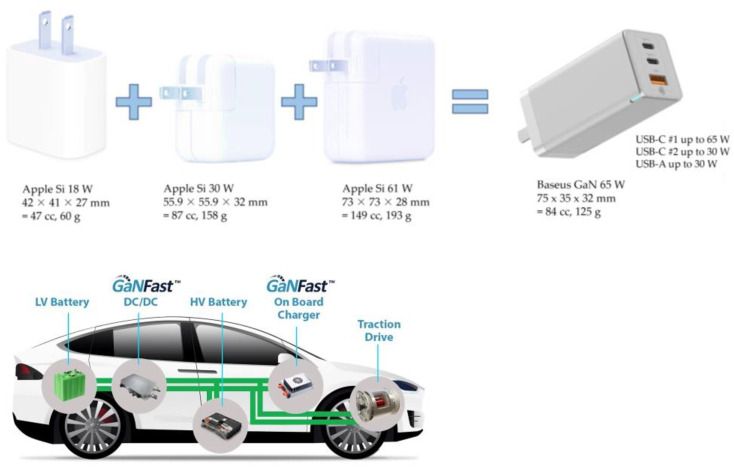
GaN technology in various power sectors [138].

Servers in data centers are also using GaN power semiconductors. Si’s capacity to handle electricity effectively and efficiently encounters ‘physical material’ barriers as data center traffic increases. Consequently, high-speed GaN integrated circuits (ICs) replace the outdated, sluggish Si chip. Major gains in efficiency are made possible by the consolidation of data center technology, a novel HVDC design strategy, and the well-proven dependability of highly integrated, mass-produced GaN power ICs. Global Si-to-GaN data center upgrades are predicted to cut energy loss by 30–40%, resulting in over 100 TWhr and 125 Mtons of CO_2_ emissions saved by 2030, shown in Figure 11 [138]. Therefore, using GaN marks another step toward the data center industry’s carbon net-zero aspirations.

GaN is becoming the preferred technology in the automotive sector for power conversion and battery charging in hybrid and electric cars. Inverters used in solar power installations, power conversion plans for motor drives, and other industrial applications increasingly use GaN-based power products. There has long been speculation that GaN will eventually replace Si power transistors. The application areas of GaN are electric vehicles, high-speed railways, household appliances, industrial motors, aerospace, smart grid, solar energy generation, wind power generation, and large capacity [141]. It is helpful for various RF applications, including satellite communications and 5G, 6G, and mobile communications, since it can operate at high frequencies, control high levels of power, and sustain a high operating voltage [177].

Moreover, GaN faced increased competition as a result of recent developments in Si SJ technology and the introduction of SiC power MOSFETs. In light of this, a reappraisal is necessary, especially in the 600 V domains involving all three [16]. The zero reverse recovery charge of lateral GaN HEMTs is one of its distinguishing characteristics. When the drain voltage drops below the total of the gate potential and the threshold voltage, a reverse channel is formed since there are no p-n junctions and current flows in a polarization-induced 2DEG [16]. Because of this property, GaN HEMTs are the best option for applications requiring continuous switching on a reverse-biased device, such as in half-bridge or full-bridge topologies. The suitability of GaN HEMTs for dual-gate structures to achieve bidirectional blocking and conducting devices [178] or to integrate multiple power devices on a common die, which makes it easier to achieve higher integration levels than with discrete components, is another intriguing feature of these devices [179].

### 4.6. Challenges of GaN Power Device

There are still various difficulties to overcome in order to replace Si technology and become mainstream. By raising the switching frequencies, GaN transistors can produce power-switching systems that are extremely compact and highly efficient [180]. GaN devices are now making excellent strides and making their way into the market, but several issues must be addressed. Vertical GaN power devices with breakdown voltages greater than 5 kV are possible as a result of advancements in substrate technology and field engineering optimization. It is also necessary to examine how the breakdown mechanism in these devices came to be [181]. Due to its potential to revolutionize power electronics with increased efficiency, better power densities, and quicker switching rates compared to conventional Si devices, GaN power devices have attracted a lot of interest in recent years. However, the development of GaN power devices faces the following major obstacles.

#### 4.6.1. Material Growth

**Figure 12:**
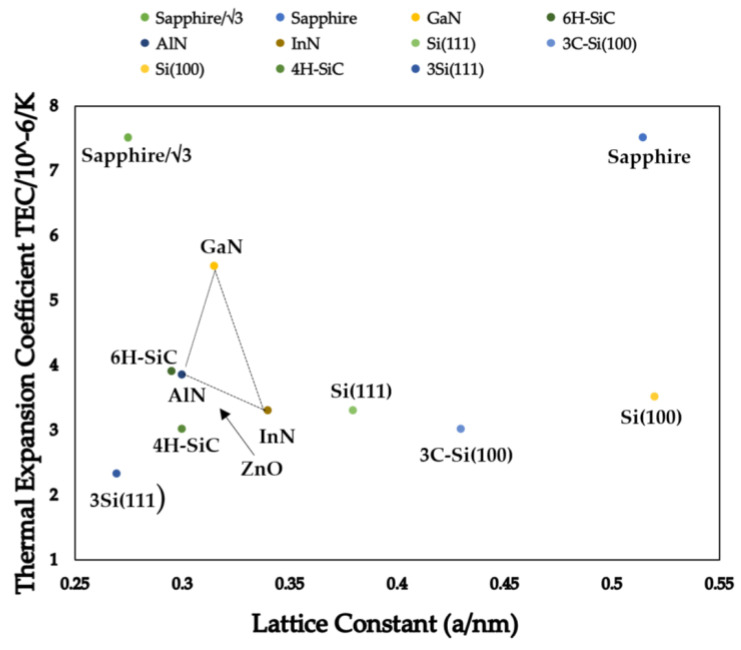
The thermal expansion coefficient of GaN and common substrates as a function of lattice constant.

GaN-based power devices are built on high-quality epitaxial material. SiC and sapphire have a smaller lattice misfit and greater thermal conductivity than Si, critical benefits for high-power devices [181]. Although the lattice mismatch between Si and GaN is substantial, its cost is modest, and the lattice mismatch can be mitigated by introducing a buffer layer for stress management, which restricts its applicability. The relationship between the thermal expansion coefficient of GaN and common substrates is shown in Figure 12 as a function of the lattice constant.

#### 4.6.2. Suppression of Current Collapse Effect

**Figure 13:**
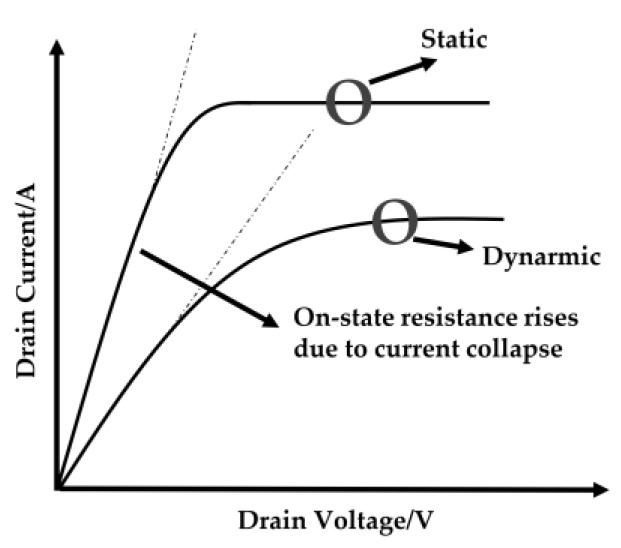
Current collapse effect.

The current collapse effect of AlGaN/GaN HEMT devices poses a severe threat to GaN power device success. It is also one of the biggest issues with today’s GaN power devices. As illustrated in Figure 13, when a significant bias is given to the drain, the leakage current degrades [182,183]. At present, the mechanism of the current collapse effect of GaN devices largely includes the following types:

1. The current collapse is triggered by carrier traps caused by deep-level centers in the material.
2. The 2DEG concentration in the AlGaN/GaN conductive channel decreases due to the change in polarization charge generated by the surface state and the surface effect, resulting in current collapse.
3. Because the material structure and the energy band structure boundary are so essential, even a minor disruption will cause the 2DEG to collapse [184,185].

#### 4.6.3. Packaging

GaN power devices have garnered considerable attention in recent years due to their exceptional performance compared to traditional Si-based devices. These devices offer superior power density, rapid switching speeds, reduced on-resistance, and enhanced thermal conductivity. Appropriate packaging solutions are essential for handling their distinctive properties and optimize their performance to ensure their efficient and dependable operation. Packaging plays a vital role in safeguarding GaN power devices against external factors like moisture, temperature fluctuations, mechanical strain, and electrical interference. It also facilitates efficient heat dissipation and establishes electrical connections with the device. A common approach for packaging GaN power devices involves utilizing surface-mount technology (SMT) packages [186]. These packages typically employ ceramic or plastic materials with a metal lead frame or solder balls for electrical connections. The package design comprises a die attach area where the GaN power device chip is mounted, bond wires or flip-chip connections to establish electrical connections, and thermal vias or heat sinks to enhance heat dissipation.

The package encompasses a ceramic or plastic body with metal leads or solder balls for electrical connections. The GaN device chip is affixed to the die attach pad at the package’s center. Electrical connections between the chip and the leads or solder balls are established using bond wires or flip-chip connections. The package may also incorporate heat sink structure to augment heat dissipation. Package designs can vary depending on the specific application and power requirements. For high-power applications, packages with larger thermal vias, enhanced heat sink structures, and improved thermal interface materials may be employed to manage increased power dissipation effectively.

Furthermore, the ongoing exploration of packaging technologies for GaN power devices aims to further enhance their performance. This involves the development of advanced materials with superior thermal conductivity, innovative interconnection like copper clip bonding or direct copper bonding, and novel package designs to minimize parasitic inductance and capacitance. Moreover, the packaging of GaN power devices is crucial for their development, ensuring reliability, efficient thermal management, and optimal electrical performance. Continued innovation in packaging techniques aim to overcome unique challenges and unlock the full potential of GaN power devices in various applications, including power electronics, automotive systems, and renewable energy.

#### 4.6.4. Gate Driver

The gate drive function assumes a critical role in the operation and performance of GaN power devices, exerting control over device switching by supplying the appropriate voltage and current signals to the gate terminal of the GaN transistor. It is imperative to meticulously design the gate drive circuitry to ensure the efficient and dependable operation of GaN power devices. Considerations related to the gate drive for GaN power devices are outlined as follows:

1. Voltage and Current Levels: GaN power devices typically necessitate higher gate voltage levels in comparison to conventional Si-based devices. Operating with gate voltages ranging from 6 V to 10 V or even higher, the gate drive circuitry must be capable of generating and sustaining these elevated voltage levels. GaN power devices exhibit low gate capacitance, enabling faster switching but demanding careful attention to the gate driver’s current capability.
2. Gate Driver ICs: GaN power devices often employ specialized gate driver integrated circuits (ICs). These ICs are specifically designed to deliver the required voltage and current levels, incorporate protection features, and enhance the overall performance of GaN transistors. Protection features may include under-voltage lockout (UVLO), over-current protection, and short-circuit protection to ensure device safety during operation.
3. High-Speed Switching: GaN power devices are renowned for their rapid switching speeds, which can present challenges in gate drive design. To achieve optimal performance, the gate drive circuitry must be capable of providing high-speed rise and fall times to minimize switching losses. This necessitates meticulous consideration of the gate driver’s output impedance, gate trace layout, and the impact of parasitic elements in the gate circuit.
4. Gate Resistance: The appropriate selection of gate resistance is crucial for GaN power devices. A suitable gate resistor aids in dampening ringing effects and mitigating the risk of oscillations in the gate voltage waveform. It also limits current during switching transitions to prevent excessive power dissipation. The value of the gate resistor should be carefully optimized based on the specific GaN device and application requirements.
5. Gate Layout and Layout Considerations: A well-designed gate layout is essential in order to minimize parasitic inductances and capacitances that can negatively impact device performance. This entails keeping gate traces as short as possible, reducing loop areas, and employing techniques like guard rings and vias to manage parasitic effects. An optimized gate layout contributes to faster switching speeds, lower losses, and overall improvement in device performance.

Table 16 compares features, including split outputs, bootstrap voltage control, and target applications for five common GaN driver ICs from top manufacturers that drive GaN FETs in half-bridge designs. With configurations ranging from general purpose to automotive-qualified, the drivers offer crucial gate drive isolation and enhanced GaN switching performance as GaN transistors become more prevalent in power supply and upcoming electric vehicle applications [187,188]. The advantages of GaN FETs’ high-frequency capability and efficiency can be maximized with the right choice of GaN driver.

#### 4.6.5. Electrical Insulation

**Figure 14:**
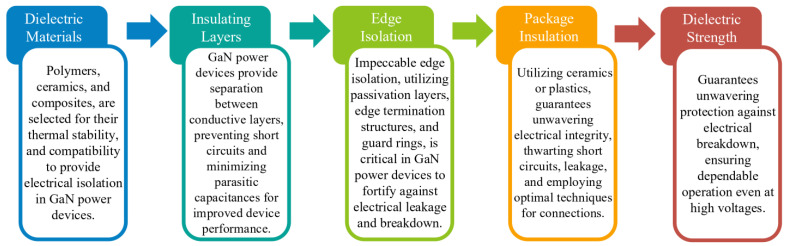
Challenges associated with electrical isolation for GaN devices.

In order to maintain adequate electrical isolation between various components, avoid electrical leakage, and improve the device’s overall dependability and safety, electrical insulation is a crucial component in the development of GaN power devices. Figure 14 illustrates challenges associated with electrical isolation for GaN devices. Barriers that withstand high voltages and prevent accidental electrical hookups are made using insulation materials and procedures. As it guarantees dependable operation and provides protection from electrical failure, electrical insulation is essential for the development of GaN power devices. Dielectric materials having sufficient dielectric strength, thermal stability, and compatibility are used to create insulation between conductive components. Insulation layers, edge isolation techniques, and the proper packaging insulation are utilized to prevent short circuits, lessen parasitic effects, and maintain electrical integrity. By giving effective electrical insulation first importance, GaN power devices may increase performance and operational reliability in many applications.

#### 4.6.6. Electromagnetic Interference

GaN power device development is heavily concerned with electromagnetic interference (EMI), as these devices can produce high-frequency switching signals that could travel as undesired electromagnetic emissions and interact with other electronic systems. To ensure the proper operation of GaN power devices and avoid interference with nearby devices or delicate electronic equipment, EMI management is essential.

1. EMI Sources: Fast switching transitions caused by GaN power devices result in high-frequency harmonics and transient currents. These may produce electromagnetic emissions that spread through the design of the device, the circuit traces, and the connections to the outside world. During switching events, voltage peaks, ringing, and current loops are the main EMI causes in GaN power devices.
2. EMI Mitigation Techniques: Several techniques are employed to mitigate EMI in GaN power devices. These include:
  - Filtering: The use of passive components such as capacitors, inductors, and ferrite beads to suppress high-frequency noise and attenuate unwanted harmonics.
  - Shielding: Incorporating shields, conductive enclosures, or grounded metal layers around sensitive components to contain electromagnetic fields and prevent their propagation.
  - Layout Optimization: Carefully consider trace routing, component placement, and grounding techniques to minimize loop areas, reduce parasitic inductance, and control impedance.
  - Grounding and Bonding: Establishing proper grounding and bonding practices to minimize ground loops, reduce voltage differentials, and provide an effective return path for high-frequency currents.
3. Compliance with EMI Standards: GaN power devices must adhere to particular electromagnetic compatibility (EMC) requirements to ensure their functioning is below acceptable EMI limits. Evaluations of radiated emissions conducted emissions, and vulnerability to outside electromagnetic fields are all part of compliance testing.
4. EMI Filtering Components: Choosing the right EMI filtering components is essential when creating GaN power devices. These parts must be tuned for the device’s working frequency range, have high-frequency filtering capabilities and low parasitic elements, and be parasite-free.
5. EMI Simulation and Modeling: EMI behavior in GaN power devices may be predicted and examined using simulation and modeling techniques throughout the design process. Using these technologies, engineers may reduce EMI problems by optimizing circuit design, filtering tactics, and grounding procedures.

### 4.7. Future Trend of GaN

Due to the relatively recent industrial introduction of GaN, future developments are a crucial topic of discussion when examining the potential uses of this technology in various applications [189]. The high cost of these devices, the current GaN devices’ limited voltage rating, the complex gate driver design and control complexity, the area-specific thermal resistance in GaN-based IC development, and packaging issues to ensure these devices’ long-term reliability present the biggest challenges and areas for improvement. Below, each of these elements is explored to determine where GaN’s future lies [190]. However, GaN will ultimately replace old Si in data centers, home solar energy systems, and other consumer applications like fast chargers and other consumer applications. GaN technology will be used more frequently in electric vehicles’ onboard chargers [191].

#### 4.7.1. Cost Reduction

GaN devices can be manufactured on Si substrates, a standard industry procedure today, to address the cost issue connected with GaN. GaN’s attractive material features and the development of fabrication facilities that are compatible with complementary metal-oxide semiconductors (CMOS) work together to provide power devices that perform better and are more affordable. Increased demand for power converter applications may result from the development of high-power ICs using GaN on Si wafers, which can further lower these costs [192].

As semiconductor technology has advanced since the commercialization of GaN technology, the unit cost of these transistors has dropped considerably. For instance, manufacturers like GaN Systems now sell the 650 V, 15 A e-mode GaN for around $12, whereas, formerly, a standalone GaN MOSFET had a unit price of about $75. This demonstrates that, as more power electronics applications adopt GaN technology, the cost is anticipated to drop further during the ensuing years due to economies of scale. GaN HEMTs can be widely used in power electronics due to their low cost, which greatly improves the performance and efficiency of electrified transportation mediums, lowering the entire system cost. Therefore, GaN HEMTs are more advantageous than they are expensive, effectively resulting in cost savings for production and operation [190].

#### 4.7.2. Thermal Management

The vertical GaN devices feature higher breakdown voltage and current than the lateral GaN designs without growing the chip’s size. Vertical structures are more dependable. In addition, vertical GaN devices have easier thermal management than lateral ones [190]. Vertical GaN structures have many advantages, and a lot of research and development is being carried out to make these structures commercially viable. When working with GaN devices, thermal management is a crucial concern. In the development of GaN-based ICs, minimizing area-specific thermal resistance is crucial. Future GaN device technologies are anticipated to use diamond and SiC substrates with strong heat conductivity [191]. The terminals of lateral GaN on Si substrate devices are on the same side of the die.

As a result, these chips have bumps added to them for mounting purposes. These bumps only cover a small percentage of the die surface area and have a low thermal conductivity. Either through these bumps, known as topside cooling, or through their Si substrate material, known as backside cooling, the GaN transistors are cooled. The top side of the die has the greatest thermal performance for thermal dissipation [191]. Future designs of highly competitive power electronic converters might include a phase-leg power module based on GaN devices that incorporates the power stage, the gate driver control circuitry, and the cooling system into a single container based on the present integrated modules [191]. The combined module will have enhanced thermal management and high-power capacity. These modules might address some of the problems that electric transportation is now experiencing, as previously mentioned [189].

#### 4.7.3. Gate Driver Design

The first GaN power IC Process Design Kit (PDK), which is known as All GaNTM, enables the monolithic integration of 650 V GaN IC circuits with GaN MOSFETs. The integration of the GaN driver with the GaN MOSFET is crucial for high-frequency operation. Due to its fast-switching transitions, the discrete GaN’s gate is susceptible to noise and voltage spikes, which can lead to damage. Noise can be reduced to some extent by integrating the GaN MOSFET with the driver. However, the GaN MOSFET’s inclusion in a multi-chip module is not without its difficulties, as larger losses are caused by the impedance between the GaN MOSFET’s gate and the Si driver output. The best possible efficiency, speed, and robustness can be attained with monolithic integration [189]. Since the driver is integrated, one module can contain the logic circuitry, startup protection, dv/dt control, and dv/dt robustness. Two MOSFETs are combined with the driving and protection circuitry in half-bridge power ICs. The level-shifter losses are 10 times lower with the 650 V GaN power IC than Si [190]. The development of more efficient, more powerful, and less expensive power systems will be made possible by next-generation monolithic integration, which includes enhanced I/O features, over-current, and over-temperature protection [190].

**Figure 15:**
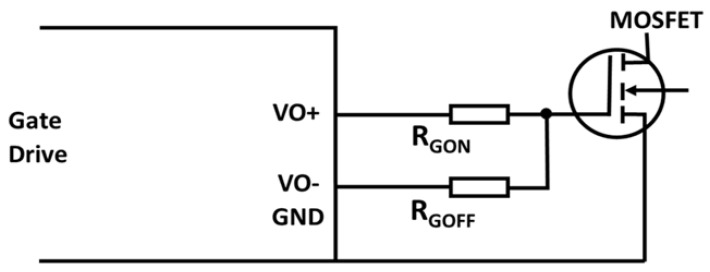
GaN E-HEMT gate driver.

In the near future, it is anticipated that gate drivers with inbuilt short circuit protection will be developed. This is particularly helpful in applications that adopt a modular approach, in which case the size of the parallelized packages is crucial. For instance, aircraft DC/DC converters are frequently built using the modular technique. The size of the power electronic components designed for industry continues to shrink as a result of advances in semiconductor technology. More power-dense DC/DC converter designs are possible with integrated gate drivers. GaN HEMT gate drive design calls for meticulous design considerations. For turn-on and turn-off, a separate gate resistor is usually advised. This is significant because the RGON has control over the dv/dt slew rate. The turn-on gate resistor’s value must be carefully chosen because, if it is too high, switching will be slowed down and result in larger switching losses, and, if it is too low, gate oscillation will cause more switching losses. It is suggested that the turn-off gate resistor, RGOFF, for GaN System’s GS66508 be between 10 and 20ω. RGOFF enables quick pull-down for a powerful gate drive and starts from 1 to 2ω. Figure 15 depicts a general layout of the gate driver schematic [193].

#### 4.7.4. Motor Drive

Because of its exceptional features, GaN has been suggested as a legitimate substitute for conventional Si-based MOSFETs and IGBTs in the motor control field. GaN technology offers effective, light, and low-footprint solutions with up to 1000 times the switching frequency of Si and lower conduction and switching losses. GaN power transistors have a switching speed that can reach 100 V/ns, which allows engineers to employ inductors and capacitors with lower values and smaller sizes [194]. Low R_DS_(on) improves energy efficiency and enables more compact dimensions by reducing the amount of heat produced. GaN-based devices require capacitors with greater working voltages, higher dV/dt transient tolerance, and lower equivalent series resistance [194] than Si-based devices do. GaN has a high breakdown voltage (50–100 V, as opposed to the typical 5 to 15 V values attainable with conventional semiconductors), enabling power devices to run at greater input energies and voltages without harm. This is an additional benefit that GaN offers. It may reach a wider bandwidth with higher switching frequencies, which enables the implementation of motor control algorithms with greater precision. Additionally, it is possible to attain a degree of efficiency with variable frequency drive (VFD) motor control that is not possible with ordinary Si MOSFETs and IGBTs. Subsequently, because the motor speed can be ramped up and down, the load may be maintained at the desired speed, and the VFD achieves an incredibly accurate speed control. Because GaN transistors switch significantly more quickly than their Si counterparts, parasitic inductances and losses are reduced, switching performance is improved (less than 2-ns rise and fall time), and the heat sink can be reduced in size or even eliminated. Very low switching losses in the GaN power stage enable greater pulse width modulation (PWM) switching frequencies with a peak efficiency of up to 98.5% at 100 kHz PWM [194].

#### 4.7.5. 5G

GaN can amplify high-frequency signals (even those of the order of a few gigahertzes) very effectively, which opens up several real and exciting opportunities in the RF industry. Thus, it is feasible to develop high-frequency amplifiers and transmitters that can travel great distances and have uses in base stations, satellite communications, radar, early warning systems, and other technologies [194]. In terms of increased capacity and efficiency, reduced latency, and all-around connectivity, 5G offers substantial advantages as the next-generation mobile technology. It is necessary to employ materials like GaN that can give high bandwidth, high power density, and excellent efficiency values for the use of various frequency bands, including the sub-6-GHz band and the millimeter-wave (mmWave) (above-24-GHz) band [194].

Due to its physical characteristics and crystalline structure, GaN can allow higher switching frequencies than comparable laterally diffused MOSFET devices at the same applied voltage, resulting in a substantially smaller footprint. RF front-end chipsets are necessary for emerging 5G technologies like massive multiple-input multiple-output (MIMO), and mmWave. The greatest option for high-power 5G and RF applications is GaN-on-SiC, which combines the high power density of GaN with the excellent thermal conductivity and low RF losses of SiC. There are currently a number of GaN-based products appropriate for 5G applications on the market, including multiple channel switches and low-noise amplifiers for massive 5G MIMO applications [194].

#### 4.7.6. Data Centers

In the data center industry, where high performance and cost reduction are fundamental concerns, the marriage of GaN with Si also presents significant prospects. Voltage converters are frequently used in data centers, where cloud servers run continuously, with typical values of 48 V, 12 V, and even lower voltages for powering the multiprocessor system cores [194]. Power-conversion efficiency has emerged as a crucial consideration for businesses looking to achieve net-zero carbon emission, especially those running data centers and providing cloud computing services. This is due to the constantly rising worldwide electricity generation. GaN technology may significantly address the need for more power in less space for data centers by attaining improved efficiency in converters and power supply, size reduction, and better thermal control, which lowers costs for the provider [194]. AC/DC converters are quite prevalent in data centers, which first regulate the bus voltage to a DC value using a power factor correction (PFC) front-end stage. Next, a DC/DC stage steps down the bus voltage and produces a galvanically isolated and controlled DC output (48 V, 12 V, and more). The PFC stage maximizes real power [194] by keeping the power supply’s input current synced with the main voltage.

## 5. Ultrawide Bandgap Semiconductor

AlGaN/AlN, diamond, and Ga_2_O_3_ are examples of ultrawide bandgap (UWBG) semiconductors, which have bandgaps that are substantially wider than those of traditional wide bandgap materials like SiC and GaN [195]. Due to their extraordinarily wide bandgaps, which allow for properties like high breakdown voltages, high operating temperatures, and high-power densities, these materials have the potential to lead to significant advancements in electrical and optoelectronic devices. In contrast to more established semiconductors like gallium arsenide and Si, UWBG materials are still in a very early stage. In areas like substrate availability, doping, comprehending carrier transport mechanics, and constructing useful devices, considerable obstacles need to be addressed.

As the bandgap increases, the figures of merit for devices like power switches scale positively, indicating that UWBG materials could permit improved performance. However, UWBG materials are still in their infancy and face formidable obstacles in areas including material production, doping, substrate accessibility, and fundamental physics comprehension. This article examines the current state-of-the-art and points out important areas for future research in material growth, physics, devices, and applications.

Creating large-diameter native substrates, comprehending growth dynamics, and attaining controlled doping, particularly p-type doping, are important issues in the field of materials. The creation of innovative UWBG materials such as BN alloys offers opportunities. To describe high-field carrier dynamics in UWBG materials, new transport physics models are required. It is also difficult to confine carriers using UWBG heterostructures. Opportunities exist in both TCAD modeling and thermal transfer. The need for advancements in vertical device topologies, contacts, packaging, and other areas makes applications like high-voltage power electronics, RF and microwave devices, deep UV optoelectronics, and severe environment electronics intriguing.

An overview of the advancement of semiconductors based on diamond from the 1980s to the present is shown in Table 17. It follows the development of early research devices, such as Schottky diodes built of single-crystal diamond, through more sophisticated polycrystalline diamond devices, such as vertical MOSFETs and lateral MOSFETs. The table lists each device’s significant accomplishments, technical details, and features.

Diamond bipolar transistors, diamond Schottky diodes, and diamond MISFETs are three different categories of diamond-based semiconductor devices that are compared in Table 18. The advantages of diamond’s high bandgap, high critical electric field, high thermal conductivity, and high breakdown voltage are shared by all three devices. Moreover, Table 18 highlights diamond devices’ high-voltage, high-power, high-temperature, and high-frequency working capabilities.

## 6. Current Innovation and Comparison

WBG and future UWBG semiconductors will be used to replace conventional Si power devices in the new generation of power devices for power converters. The commercial Si IGBT dominant power switch’s current maximum breakdown voltage capacity is 6.5 kV. In any case, a Si-based gadget could not function above 200 °C. These inescapable physical restrictions severely reduce the efficiency of modern power converters, necessitating, among other things, complicated and expensive cooling systems [196]. These novel power semiconductor materials can make electric energy transformations more efficient, leading to a more judicious use of electricity and a smaller carbon impact.

SiC and GaN are the most desirable candidates among the WBG semiconductors because they already offer a good compromise between their theoretical properties (blocking voltage capability, operation temperature, and switching frequency) and commercial presence [15]. Both are ideal candidates to replace Si in the following wave of high-power and high-frequency electronics due to their wide bandgaps, resulting in higher breakdown voltage and operation temperature than Si [197].

Electrical quantities like voltage, frequency, and operating temperature define the application of the power system in power electronics. The physical characteristics of Si, SiC, GaN, and diamond materials are listed in Table 19 [198,199,200]. Furthermore, Table 19 compares key physical properties and device parameters of Si, SiC, GaN, and diamond semiconductors. It shows that wide bandgap semiconductors like SiC, GaN, and especially diamond have superior attributes like higher breakdown fields, thermal conductivity, and switching frequencies compared to Si, enabling high-power and high-frequency applications. Diamond has the greatest combination of properties overall such as a very high breakdown field and thermal conductivity.

The bandgaps of WBG semiconductors are roughly three times, and the electric field values of SiC polytypic and GaN are comparable and much greater than those of Si. Higher radiation hardening and high-temperature operation are advantages for WBG semiconductors. For Si, this occurs at a temperature of about 150 °C [201], where around 900 °C is the inherent temperature of 4H-SiC, and the temperature range between 300 °C and 800 °C has been used to test manufactured AlGaN/GaN HEMTs [202]. They can be utilized in harsh environments where Si-based devices are ineffective [203].

In comparison to Si, SiC, and GaN, diamond has an extraordinarily wide bandgap of 5.45 eV and the highest electrical breakdown field, thermal conductivity, electron velocity, and current density. This makes diamond an exceptional material for creating semiconductor devices with an extremely high power density, high frequency, and high temperature. To construct useful diamond electronic devices, however, significant obstacles still exist in creating large, high-quality diamond substrates and accomplishing doping and metallization. With certain manufacturing challenges, SiC and GaN also have very wide bandgaps when compared to Si, enabling high-voltage, high-power, and high-speed devices. Overall, these ultra-wide bandgap semiconductors promise to significantly outperform Si devices in terms of performance.

In the context of electronics, certain constraints that apply to optoelectronics are relaxed. For instance, the bandgaps of these materials do not need to be direct, making SiC a viable option. Additionally, light emission efficiency is not as critical for electronics, making GaN and aluminum GaN (AlGaN) suitable materials, not just indium GaN (InGaN) [17]. Among these materials, SiC has a longer history and has seen sustained investment since the late 1970s, particularly through the U.S. Department of Defense Office of Naval Research. This investment has led to significant advancements in SiC material synthesis and quality, as well as progress in various device technologies.

**Figure 16:**
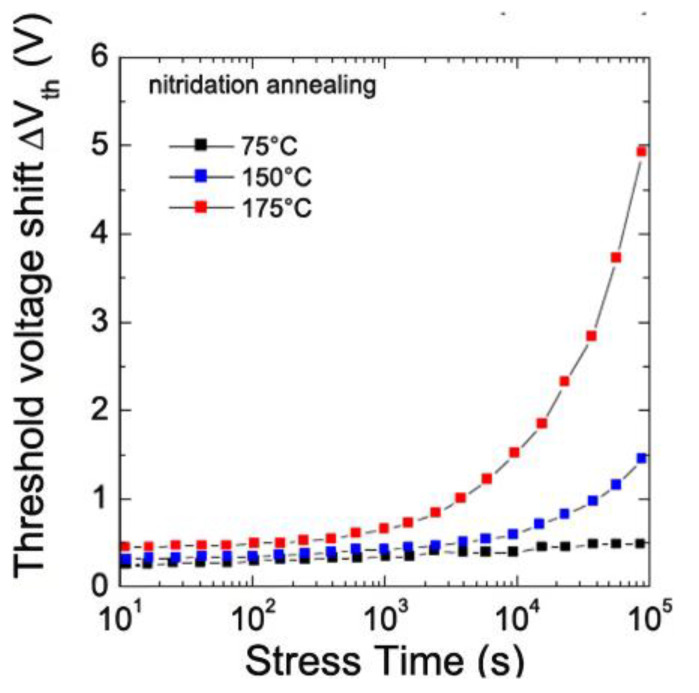
Threshold voltage shift V_th_ with relation to stress time at various temperatures [204].

Over the past few years, 4H-SiC has drawn more attention as a suitable material for high-voltage power applications. The first Schottky barrier diode (SBD) was promoted in 2001, and this marked the realization of the SiC-based power electronics goal [204,205]. Photovoltaic (PV) inverters, power supply, and power factor correction circuits (PFC) are the main applications for SiC diodes. Figure 16 shows the variation of V_th_ for a 4H-SiC MOSFET with a nitridated SiO2 layer as a function of stress time and temperature.

**Figure 17:**
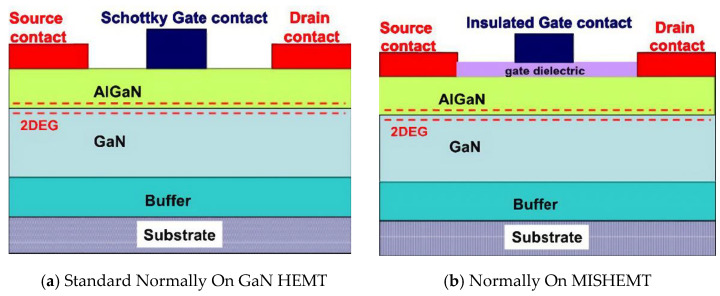
Schematic of configurations for normally on AlGaN/GaN HEMTs with (**a**) Schottky gate; and (**b**) and insulated gate [17].

Due to its sensitive gate dielectrics, the SiC MOSFET offers questionable reliability; the major issue is the comparably poor channel mobility. It is a suitable choice for the fabrication of power electronics devices with high break-down voltage, low specific R_ON_, and high-frequency switching operations due to physical properties like a high saturation velocity and a high critical electric field [206]. GaNs’ stronger critical electric field and higher electron mobility should assure substantially superior efficiency compared to SiC [15]. The GaN HEMT is inherently a normally on device because of the existence of the 2DEG, which can be seen in Figure 17a,b [17].

In general, the AlGaN/GaN heterojunction typically generates a two-dimensional electron gas (2DEG) and a high sheet carrier density (of around 10 cm) as a result of piezoelectric polarization [207]. Moreover, HEMTs are devices whose functioning is predicated on the presence of 2DEG [15,204]. Developing well-engineered normally off GaN HEMT technologies, improving insulator/GaN interfaces, enhancing efficient metallization plans for GaN-on-Si frameworks, and other issues are a few of the difficulties.

Ultrawide bandgap (UWBG) semiconductors represent an interesting and difficult new field of study in semiconductor materials, physics, devices, and applications, with bandgaps considerably larger than the 3.4 eV of GaN [17]. Furthermore, these UWBG materials have the potential for far higher performance than standard WBG materials because many figures of merit for device performance scale with the increasing bandgap in a very non-linear way. The commonly used Baliga figure of merit (BFOM) [208] is defined as V^2^_BR_/R_ON-SP_ in the straightforward situation of a low-frequency unipolar vertical power switch, for instance, where R_ON-SP_ stands for the specific on-resistance, which is the inverse of the conductance per unit area while the switch is on [208,209]. The device’s capacity to block electricity while turned off and/or its conductivity per unit area when turned on increases with increasing BFOM. The BFOM scales as about the sixth power of the semiconductor bandgap because the critical electric field scales roughly as the square of the semiconductor bandgap.

To put this trio of materials in perspective, Table 20 lists some of their physical characteristics as well as the state-of-the-art values for three metrics crucial for device applications, for instance, the quality of their substrates as determined roughly by dislocations per square centimeter and substrate diameter, their capacity for p-type and n-type doping, and their capacity for n-type doping.

Moreover, Table 20. lists some of the material characteristics of WBG and UWBG semiconductors, as well as the most recent developments in the following areas, such as substrate dislocation density, substrate diameter, and bulk p-type and n-type doping levels [17]. From an electronics perspective, they possess the following advantageous features, for example, a wide range of direct bandgaps, spanning from 3.4 eV to approximately 6.0 eV; high breakdown fields, with AlN exhibiting values exceeding 10 MVcm^−1^; high electron mobility, reaching bulk mobilities of up to 1000 cm^2^V^−1^s^−1^; high saturation velocities exceeding 10^7^ cms^−1^; and the relative ease of n-type doping with Si, which has a relatively small donor ionization energy, especially up to approximately 80–85% aluminum content [210,211].

**Figure 18:**
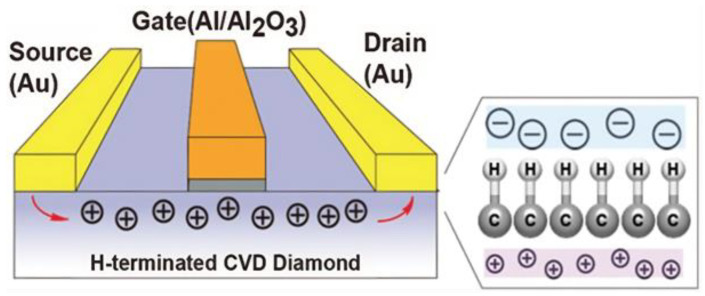
Diamond’s hydrogen termination decreases its ionization energy and promotes electron transfer from the surface’s valence band into other materials that adsorb the electrons [212].

From an optoelectronics perspective, AlGaN alloys enable the direct generation of emission wavelengths shorter than 365 nm, extending into the ultraviolet A, B, and C bands. Similarly, InGaN and AlGaN, are ternary alloys, which enables the use of heterostructures and bandgap engineering, a strategy successfully employed by other ternary alloys in the III–V materials family. UWBG applications, including high-power and high-frequency electronics, radiation detectors, electron emitters for ultra-high-voltage vacuum switches and traveling wave tube cathodes, and thermionic emitters for energy conversion, are all made possible by the exceptionally beneficial characteristics of diamond. As shown in Figure 18, lateral metal-oxide-semiconductor field-effect transistor (MOSFET) devices have now been created using atomic-layer-deposited (ALD) dielectric layers, even though these air-exposed surfaces are noticeably unstable.

Diamond also holds the highest known thermal conductivity of any material, which is particularly significant because heat removal is a major limiting factor in the performance of many power electronics and optoelectronics applications. Excellent electron emissivity on hydrogen-terminated surfaces, surface transfer doping made possible by these surfaces, [15] room-temperature UV exciton emission, and optical defect centers brought on by the nitrogen-vacancy (N-V) and Si-vacancy (Si-V) complexes are a few additional special qualities. For emerging quantum information systems, these defect centers have been proposed as a physical platform for qubits [148].

Table 21 summarizes the state-of-the-art performance ranges for their respective technologies’ evaluated powers and frequencies. It is possible to observe the trends in the applications of SiC technologies at higher powers, as the finest performance range of the technology gradually increases with the levels of the current in the transistor, and of the GaN technology at higher switching frequencies and lower power levels.

A contemporary power semiconductor device switches rapidly between the ON and OFF states. A perfect switch may switch at any frequency and have no power losses in either the ON or OFF states. Losses do occur in practical devices, primarily in the ON state and during switching transitions. Three-terminal switches are required to produce the regulated ON and OFF transitions, with the third terminal controlling the transition by either supplying a voltage or a current signal. BJTs, thyristors, and gate turn-off (GTO) thyristors are current-controlled devices, whereas MOSFETs and IGBTs are voltage-controlled switches [213].

In contemporary power electronics converters, two terminal switches with unidirectional current flow capabilities, such as a diode, are also required. In general, the voltage and current ratings are any power device’s most crucial parameters. Switching speed is another important specification that may be used to compare device capabilities. Devices with different breakdown voltage ratings are created for applications requiring a range of voltage levels [213]. Power devices with breakdown voltages above 600 V are often needed for important industrial and renewable energy applications, such as PV, wind, EV, and industry motor drives. Power devices with a voltage rating between 20 V and 600 V are commonly used for power supply applications in computers, mobile computing devices, and data centers [213]. Since the performance heavily depends on the voltage rating of the device, different devices can only be compared when they are made for the same breakdown voltage.

### 6.1. Voltage Rating

**Figure 19:**
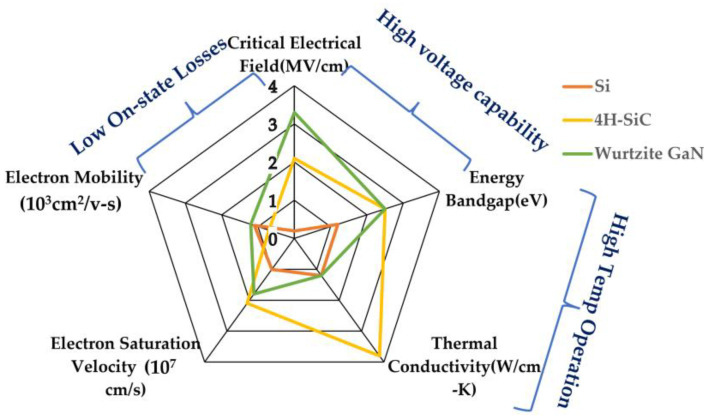
Critical material properties of Si, 4H-SiC, and Wurtzite GaN [214].

Modern power semiconductor devices are made by vertically stacking several P-type and N-type semiconductor layers on a substrate crystal wafer. The chip’s main electrical terminals are on both sides. The switch function is accomplished by altering the device’s conductivity from high in the ON state to low in the OFF state. When the maximum electric field reaches a critical breakdown field E_c_, the voltage rating is commonly specified as the breakdown voltage. The semiconductor material in question determines E_c_. A large OFF-state leakage current will be observed if the breakdown is reached electrically. The operation voltage is often chosen to be substantially lower than the breakdown voltage, due to the necessity of enduring transient overvoltage spikes, as well as the converter’s long-term stability. The critical field E_c_ for Si is around 20 V/m, while E_c_ for broader bandgap materials like SiC and GaN is close to 300 V/m. For the three materials stated, this is graphically depicted in Figure 19.

The use of wider bandgap power devices such as SiC and GaN has several advantages, including greater Ec and other desirable material features such as higher thermal conductivity. The same breakdown can be obtained in SiC material with an inner depletion thickness of less than 70 m and a surface termination region of roughly 200 m. As a result, achieving exceptionally high breakdown voltage in SiC power devices is significantly easier.

### 6.2. Current Rating

**Figure 20:**
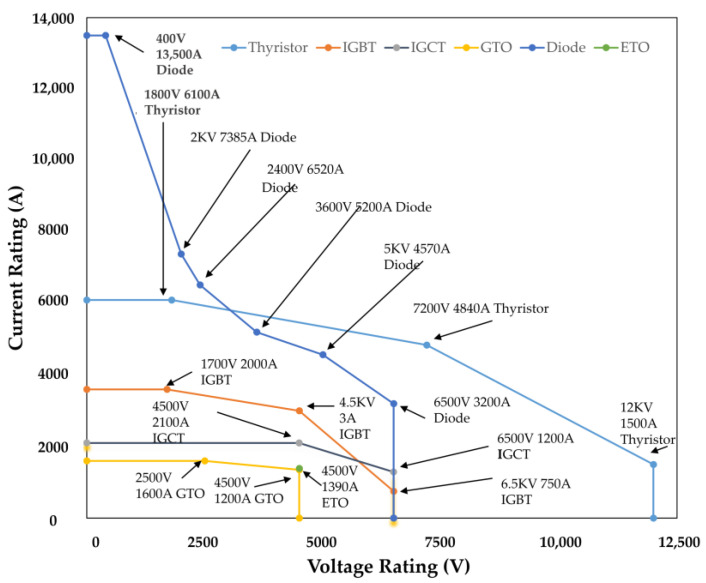
State-of-the-art commercial Si power devices in terms of the upper boundary of the voltage and current ratings achieved in a single-packaged device. The current rating shown is the DC rating at a case temperature of 85 °C.

**Figure 21:**
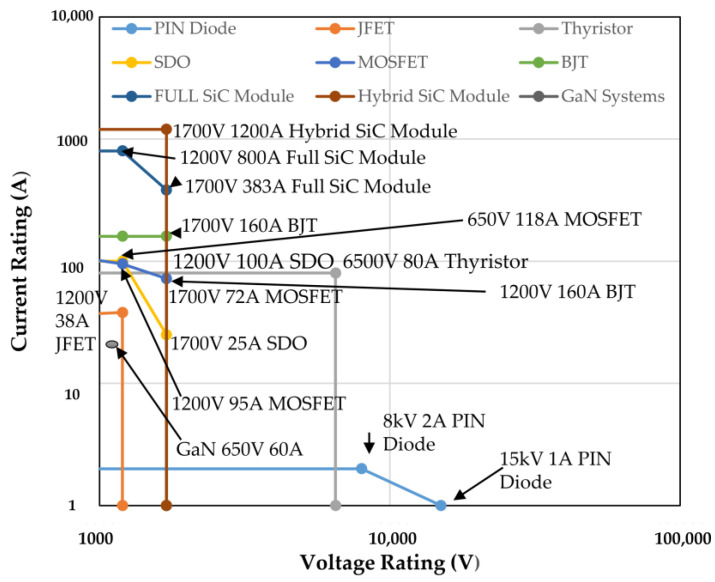
State-of-the-art commercial WBG power devices in terms of the upper boundary of the voltage and current ratings achieved in a single-packaged device. The current rating shown is the DC rating at a case temperature of 25 °C.

When the device conducts current in the ON state, the generated heat is manageable and does not cause the device to surpass its maximum operating temperature. Device innovation is motivated by increasing current density for a given breakdown voltage. Comparing absolute voltage and current ratings is one technique to assess state-of-the-art power devices, particularly their commercial readiness. This is depicted in Figure 20 and Figure 21 for commercially available Si power devices and SiC and GaN power devices, respectively. Because of the excellent bipolar conduction mechanism in these two devices, the Si thyristor and Si diode have reached the highest voltage and current ratings. These two devices are also bundled in press-pack packaging and fabricated on a single wafer utilizing the level edge termination technique [215,216,217]. SiC and GaN power devices, first introduced to the market a decade ago, have made substantial progress in terms of commercially accessible voltage and current ratings. Clearly, there is still a significant disparity in the current ratings of Si power devices, as illustrated in Figure 20. As illustrated in Figure 21, hybrid devices built by Si IGBT and SiC diodes are being presented to fill this gap [218,219].

### 6.3. Switching Frequency

The power device in modern power converters must switch at high frequencies. Switching at higher frequencies has advantages such as improved dynamic response and smaller, lighter passive components. For high-density power electronics, reducing the size of passive components is crucial. As a result, switching frequency is another crucial metric to consider when comparing power devices. The device’s switching losses during turn-on and turn-off limit the switching frequency’s upper limit. As a result, the switching frequency is a tradeoff between conduction and switching loss, rather than a theoretical restriction on how fast the device can flip. Huang’s thermal figure of merit (HTFOM) in Table 22 can also be used as the switching frequency figure of merit (FOM). It indicates that hard-switching WBG switches will be limited in their frequency by poor thermal conductivity and/or small chip size [220]. Likewise, their chip size reductions have fallen short of the Huang chip area figure of merit (HCAFOM) prediction in Table 22.

Table 23 indicates that die size decreases significantly when technology advances from standard Si MOSFETs to SJ MOSFETs to SiC. SiC MOSFETs are around 20 times smaller than Si MOSFETs. The size reduction in GaN is less significant than in SiC since the GaN device is a lateral power device rather than a vertical power device, and its R_ON-SP_ decrease is lower. The greater die size, on the other hand, offers superior thermal performance. The lateral GaN has the added benefit of having a reduced capacitance/gate charge. The lateral structure is to blame for this. The SiC and GaN power devices are well-positioned to operate at higher frequencies in hard-switching or soft-switching converters due to significant reductions in DFOM1, DFOM2, and DFOM3.

### 6.4. Thin Wafer Field Stop IGBT (FS-IGBT)

IGBT technology became the essential notion virtually as soon as the planar power MOSFET was presented [221]. They are available in single switch and rotor configurations, with ratings ranging from 250 A to 1200 A. In motor control and drives, uninterruptible power supplies (UPS), transmission and distribution, commercial, construction, and agricultural vehicles (CAV), as well as traction uses, 4500 V and 6500 V IGBT modules are frequently used [222]. As a result, MOSFETs, GTOs, and BJTs have been rapidly phased out of medium- to high-power applications. In terms of technology, three decades of invention and industrialization have introduced various generations of IGBT technology. The implanted P collector is no longer reached by the depletion region. E_off_ may be modified due to the implanted collector, which allows for control of minority carrier injection. The non-punch through (NPT) IGBT, on the other hand, has a longer drift layer, which increases forward voltage (V_f_) once more [220].

### 6.5. Reverse Conducting IGBT (RC-IGBT)

Because IGBTs lack a reverse conduction path, an externally packaged freewheeling diode (FWD) is required to let the current flow in the other direction. The reverse recovery loss should be reduced, and the recovery softness must be improved according to key design factors. A new generation of IGBTs with inbuilt FWD has been released [223,224,225]. The thin wafer manufacturing technology established for the FS-IGBT is used in the RC-IGBT. An N region is created by interrupting the backside P collector. The RC-IGBT chip can now take up the entire module footprint in an RC-IGBT power module. The MOS-controlled diode investigated this MOS control characteristic many years ago [226,227,228].

### 6.6. Reverse Blocking IGBT

To inhibit reverse voltage, certain significant renewable energy converters, such as the T-NPC three-level converter [229], need an IGBT in series with a diode. Changes in the collector junction doping concentration and edge termination must be performed to enhance the voltage. Reverse blocking IGBT (RB-IGBT) is one such RB-IGBT [230]. The N buffer layer must be removed to enhance the reverse breakdown voltage, converting the IGBT to an NPT-IGBT. To minimize the surface/edge electric field in the reverse direction, a new termination will be required. Deep diffusion or epitaxial regrowth following a deep etch can produce the latter.

### 6.7. Integrated Gate Commutated Thyristor (IGCT)

Because of the clear advantages of constructing a high-power device in a single device wafer and the extremely dependable press-pack packaging process, the Si thyristor, or SCR, has been and continues to be the most powerful semiconductor switch ever created. Until high-power IGBTs superseded megawatt gate turn-off (GTO) converters operating at a few hundred hertz, the high-power industry was dominated by megawatt GTO converters. The GTO’s bad turnoff safe operation area (RBSOA) is one explanation for this. In the late 1990s, a substantial advancement was made to revitalize GTO technology. The GTO’s gate drive circuit was the center of the innovation. The device begins to turn off when the current reaches about a third of the anode current. The thyristor action is still active because there is roughly a 2/3 current in the cathode/emitter junction currently. ABB has recently enhanced the IGCT’s capabilities by incorporating an inbuilt freewheeling diode into the same wafer, resulting in a reverse conducting IGCT (RC-IGCT) [231]. The emitter turn-off (ETO) thyristor [232] aims to achieve unity gain turn-off. It is possible to accomplish a 5000 A snubber-less turnoff [233]. In case of emitter turn off thyristor (ETO), built-in current sensing is similarly simple to create, and the ETO, on the other hand, is currently not in commercial production [234].

### 6.8. Reliability and Application

Reliability and applications are critical factors to take into account in order to fully utilize wide bandgap power devices such as GaN and SiC in real-world systems. Significant improvements in power density, efficiency, and high-temperature operation are made possible by these devices. Robustness under dynamic switching conditions can be impacted by problems such as current collapse, threshold voltage instability, gate oxide breakdown, and electromigration. To reduce the negative effects on lifetime, adequate characterization and derating are required in addition to methods like gate drive optimization, sophisticated packaging, and layout strategies. For example, surface passivation and buffer layer modifications can reduce on-resistance degradation caused by current collapse [235]. By using field plates and optimizing dielectric thickness, gate reliability can be increased. GaN dies, substrates, and solders’ acoustic mismatch cause stresses and defects during heat cycling, necessitating package co-design and modeling.

Utilizing wide bandgap capabilities in applications such as data center power supplies, naval electrical systems, EV charging, and renewable integration necessitates a comprehensive analysis covering device physics, packaging, thermal management, and system architectures. Wide bandgap devices are highly valuable when used in high-performance power electronics equipment because of their superior attributes such as faster switching, lower losses, and high-temperature capacity. However, their effective deployment in these devices requires a thorough understanding of degradation mechanisms, customized design strategies, and extensive qualification testing [16]. However, to do this, you need a multidisciplinary team with knowledge in materials science, accelerated testing, circuit design, application engineering, and device fabrication. Wide bandgap potential can be unlocked by holistic solutions, which also guarantee enough robustness to enable a seamless technology transfer.

Since the 1950s, Si has been the primary semiconductor substrate used in the production of power electronics equipment. On the other hand, the maximum theoretical efficiency of Si-based power-switching devices has been achieved [236]. High power losses, low switching frequencies, and decreased performance at high temperatures are some of the disadvantages of Si-based devices. As the need for distributed energy resource (DER) integration and urban electrification grows, a new class of advanced materials called wide bandgap (WBG) semiconductors has emerged. Among them are SiC, diamond, GaN, aluminum nitride (AlN), boron nitride (BN), zinc oxide (ZnO), and gallium oxide (Ga2O3). These materials have a great deal of potential for the upcoming power conversion technologies. Unipolar devices, such as MOSFETs, have limited rated voltage and current capabilities, but they can achieve high switching frequencies.

**Figure 22:**
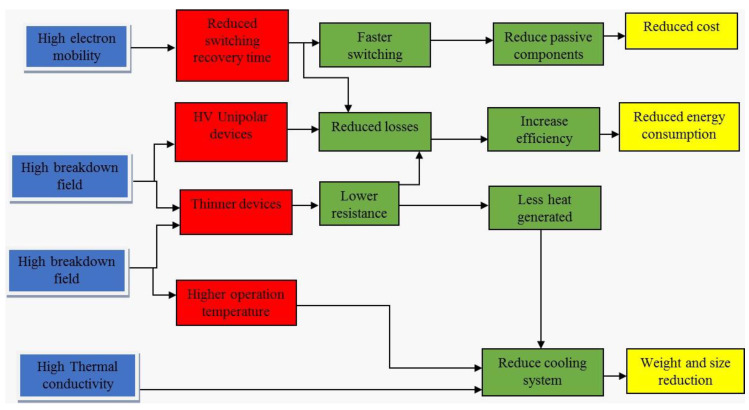
Flow of the characteristics of WBG semiconductor devices in terms of the parameter capabilities (blue); physical properties affected by WBG advantages (red); power electronics characteristics (green); and product benefits (yellow).

Bipolar Si semiconductors, on the other hand, enable the operation of high-power conversion devices at relatively low frequencies, but this requires larger and heavier passive components, which might not be appropriate for applications like power supplies, motor drives, automotive, and aerospace that have strict weight and volume limitations. Figure 22 illustrates how WBG materials show a compelling alternative to the limitations of Si by offering enhanced properties. These materials have higher electric breakdown fields, deeper doping concentrations, and thinner layers. These properties enhance their ability to block voltage and decrease drift resistance, which, in turn, reduces conduction losses. This implies that smaller WBG devices with the same on-resistance can have lower capacitance. A high saturation drift velocity allows for higher switching speeds with reduced capacitance because less energy is lost during each switching cycle [53]. Additionally, strong high-temperature performance and a reduction in leakage currents are guaranteed by the low intrinsic carrier concentration of WBG materials [237].

All the characteristics of WBG materials make them prominent semiconductor devices in high-output power equipment with increased efficiency, as well as smaller, lighter, and less expensive systems [238]. With small switching losses, WBG semiconductors can achieve 99% efficiency. When compared to Si devices, this indicates a reduction in energy losses of up to 75% [128]. Furthermore, it is possible to obtain higher switching frequencies. Due to Si limitations, frequencies higher than 20 kHz have not yet been achievable at power levels greater than tens of kilowatts; as a result, WBG materials provide better output quality and enable simpler circuit topologies by reducing the size and number of passive components [239].

### 6.9. Current Marketplace Scenario

A transformational phase is now taking place in the market environment for power electronic semiconductors, which includes Si, SiC, and GaN. Si has long dominated the industry because it is inexpensive and has reliable production methods, but SiC and GaN are emerging as disruptive technologies that are steadily capturing market share. SiC-based devices are appropriate for high-power applications like electric cars and renewable energy systems because they have higher power densities, faster switching rates, and reduced losses. On the other hand, GaN-based devices offer high-frequency operation and increased efficiency, finding use in data centers and small power converters. Si-based devices continue to rule the industry due to their maturity and widespread availability despite SiC and GaN’s increasing acceptance. In the current market environment, SiC and GaN devices are gaining ground in high-power and high-frequency applications. In contrast, Si devices continue to predominate in low- to medium-power applications.

**Figure 23:**
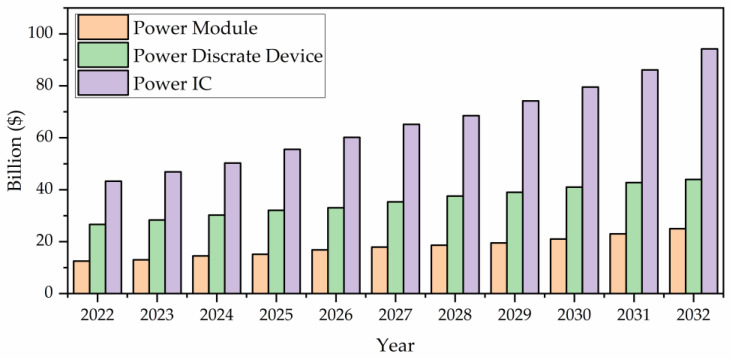
Power electronic market size projection: Will surpass USD 94.21 Billion revenue by 2032.

Power electronics are being used more often in various energy conversion end applications, propelling the considerable expansion of the worldwide power electronics market. The rising need for energy-efficient technology across a variety of end-user sectors is what drives this development. On 13 April 2023, in New York, GlobeNewswire shares [240] that, the market for power electronics will increase from USD 43.3 billion in 2022 to more than USD 94.21 billion by 2032, with a predicted compound annual growth rate (CAGR) of 8.3% from 2023 to 2032, which is shown in Figure 23.

Power semiconductor devices comprising Si, SiC, GaN, and diamond are all compared in Table 24 for their features and uses. Voltage, current, frequency, applications, packaging, features, and manufacturers are given as important parameters for each type of device and material system. The chart displays the wide bandgap materials like SiC, GaN, and diamond for power electronics’ high voltage and high-frequency capabilities.

Several commercially available power semiconductor devices from top producers like Infineon and STMicroelectronics are shown in Table 25. The table covers key characteristics of various Si, SiC, and GaN devices. These devices can be used for low, medium, and high voltage applications due to their wide voltage ratings of 20 V to 2 kV. The range of current ratings is 4.5 A to 400 A. For high-frequency GaN HEMTs, switching frequencies up to 2 MHz are mentioned. The wide bandgap SiC and GaN enable an operational temperature range of up to 200 °C. Standard packaging formats for discrete devices, such as TO-247 and TO-263, are displayed. For developing GaN technology, chip-scale packages are also listed. The chart shows how contemporary power semiconductors combine high voltage blocking capabilities, low loss switching, and high-temperature tolerance to allow performance advantages in various power electronics applications. For various applications, top manufacturers offer a wide range of Si, SiC, and GaN devices.

## 7. Conclusions

Substantial advancements have been achieved in developing novel materials like SiC, GaN, and diamond, their commercialization, and the adoption of these wide and ultrawide bandgap power electronic semiconductors. Improvements in material quality, manufacturing processes, and device performance have boosted industrial use in many application fields. The capabilities and applications of these wide bandgap technologies are continuously being expanded by ongoing research and development. Wide bandgap power semiconductors, such as SiC, GaN, and ultrawide bandgap devices like diamond technology, have the potential to revolutionize power electronics due to their superiority over Si in terms of voltage blocking, switching speeds, efficiency, and thermal performance. SiC and GaN devices are progressively increasing in market share, particularly in electric vehicles, renewable energy, aerospace, and high-frequency applications, even if Si continues to rule the market now. Diamond’s exceptionally wide bandgap could enable unprecedented power densities and high-temperature operation if manufacturing challenges can be overcome. Finally, these wide and ultra-wide bandgap semiconductors have the potential to eventually replace Si throughout the entire spectrum of power electronics due to their advantages over Si, influencing the development of next-generation, high-performance, energy-efficient devices. This article summarized the physical characteristics, difficulties, uses, and competitive environment of several prospective wide bandgap power electronic semiconductors, including the recently developed diamond technology.

## Figures and Tables

**Table 1:** Evaluation of power electronic Si-based semiconductor device.

Year	Device	Specifications	Milestone	Features
1950s	Thyristor/SCR	Up to 1000 V, 100 A	First thyristor invention	Low on-state loss
1956	Power diode	Up to 300 V, 10 A	First commercial Si power diode	Higher switching speed than selenium diodes
1958	Power transistor	Up to 60 V, 10 A	First Si power transistor	Higher gain and frequency than germanium transistors
1960s	SCR	Up to 1000 V, 100 A	Widespread SCR adoption	Phase control for AC power control
1970s	Power MOSFET	Up to 500 V, 10 A	First commercial power MOSFET	Higher switching speed than BJTs
1978	IGBT	600 V, 10 A	Invention of IGBT	MOSFET speed with BJT bidirectional capability
1980s	BJT	Up to 1200 V, 10 A	Improved high-voltage BJTs	Optimized for high-voltage applications
	MOSFET	Up to 900 V, 100 A	Trench gate and VDMOS	Reduced on-resistance
1990s	MOSFET	Up to 900 V, 100 A	Double-diffused MOSFET	Further on-resistance reduction
	IGBT	1200 V, 50 A	Trench gate IGBT	Lower losses than planar IGBTs
2000s	IGBT	6500 V, 1200 A	3rd-gen trench gate IGBT	Near ideal switching behavior
	MOSFET	900 V, 150 A	Super junction MOSFET	Low on-resistance, fast switching
2010s	IGBT	6500 V, 1500 A	4th-gen field stop IGBTs	Minimized tail current
2020s	IGBT, MOSFET	Improvement	SiC and GaN emerging	Improved, but Si is phased out for WBG

**Table 2:** Future of Multi-Si, Mono-Si, and Ribbon-Si technology.

Wafer Technology	Multi-Si	Mono-Si	Ribbon-Si
Si feedstock production (MWp)	160	-	-
Crystallization and wafer (mm)	150 × 150	125 × 125	150 × 150
Cell processing (cells)	72	-	-
Module assembly	Frameless	Framed	Frameless
Wafer thickness (µm)	285–>150	270–300	300–>200
Module efficiency (%)	13.2–>16	14–>15	11.5–>15

**Table 3:** Characteristics of selected Si MOSFETs [42].

Part Number	Current (A)	C_ISS_ (pF)	C_GD_ (pF)	R_ds(on)_ (25) (Ω)
IXFP20N50P3M	2.5	1800	7	0.3
IXFP20N50P3M	5	1800	7	0.3
IXFH16N50P3	10	1515	7	0.3
IXFR64N50P	20	9700	30	0.095
IXFR80N50Q3	40	10,000	115	0.05

**Table 4:** Specifications of Si-based semiconductor device.

Parameter	Unit	Si BJT	Si Diode	Si MOSFET	Si IGBT	Si Thyristor/GTO
Voltage rating	kV	0.8	1.2	0.6	1.2	4
Current rating	A	15	40	100	50	3000
Switching frequency	kHz	10	20	20	20	<1
Channel resistance	Ω-cm^2^	-	-	30	-	-
Off-state breakdown voltage	kV	0.8	0.6	0.6	1.2	4
Maximum junction temperature	°C	150	150	150	150	150
Conduction losses		High	Low	Medium	Medium	Low
Switching losses		Medium	Low	Low	High	High
Power losses		High	Low	Medium	High	Medium
Applications		Low voltage, low frequency	Rectification, voltage clamping	Low voltage, high frequency	Medium voltage, medium frequency	High voltage, low frequency

**Table 5:** Comparison of Si-based semiconductor devices.

Features	Si Diode	Si MOSFET	Si-SJ MOSFET	Si IGBT
Scalability	Scalable to high power levels	Scalable to medium power levels	Scalable to high power levels with parallel modules	Scalable to high power levels with parallel modules
Cost	Low to moderate cost compared to other power semiconductors	Low to moderate cost compared to other power semiconductors	Moderate to high cost compared to other power semiconductors	Moderate cost compared to other power semiconductors
Type of failure	Typically fails due to reverse breakdown, overcurrent, or excessive temperature.	Typically fails due to overvoltage, overcurrent, or overheating	Typically fails due to overvoltage, overcurrent, or overheating	Typically fails due to overvoltage, overcurrent, or overheating
Applications	Power supplies, rectifiers, freewheeling diodes, flyback diodes, voltage clamping, snubber circuits, battery charging	Switched mode power supplies, lighting, audio amplifiers, consumer electronics, automotive applications	Power supplies, solar inverters, server power supplies, industrial applications	Motor drives, industrial applications, renewable energy systems, UPSs, electric vehicles

**Table 6:** Evaluation of power electronic SiC-based semiconductor device.

Year	Device	Specifications	Milestone	Features
1990s	Schottky diode	600 V, 1 A	First commercial SiC diode	Higher voltage capability than Si
2001	JFET	1200 V, 5 A	First SiC transistor	Higher bandgap than Si devices
2006	MOSFET	1200 V, 10 A	First commercial SiC MOSFET	Higher frequency capability than Si IGBTs
2010s	MOSFET	1700 V, 100 A	Trench gate SiC MOSFETs	Reduced on-resistance
	JFET	1700 V, 50 A	Normally-off SiC JFETs	Simpler gate drive than depletion mode
	BJT	1200 V, 15 A	Higher current SiC BJTs	Improved SOA over Si BJTs
	SBD	1700 V, 20 A	Low loss SiC Schottky diodes	Faster switching than Si PiN diodes
2015	MOSFET	3300 V, 24 A	Higher voltage SiC MOSFETs	Expanding adoption in EV/PV markets
2018	IGBT	3300 V, 100 A	First commercial SiC IGBTs	Entering higher power applications
2020s	MOSFET	>10 kV, >100 A	Voltage and current increase	Replacing Si IGBTs and thyristors
Future	IGBT	>10 kV, >100 A	SiC IGBT refinement	Competing with Si IGBTs
	GaN	GaN/SiC	Combining GaN and SiC	Performance greater than either alone

**Table 7:** Characteristics of selected SiC MOSFETs [28].

Part Number	Current (A)	C_ISS_ (pF)	C_GD_ (pF)	R_ds(on)_ (25) (Ω)
IMW120R350M1H	2.5	180	1	0.35
C3M0280090J	5	150	2	0.28
IMW120R220M1H	10	289	2	0.22
C3M0120100K	20	350	3	0.12
SCT4026DE	40	2320	9	0.026
SCT4013DR	80	4580	10	0.013

**Table 8:** Electrical characteristics of SiC-IGBT and Si-IGBT devices [94].

Manufacturer	Advanced Power Technology	Semikron	Mitsubishi
Device type	SiC-IGBT	SiC-IGBT	Si-IGBT
Part number	APT60GF120JRDQ3	SK25GH063	CM150DY-24A
Used experiments	SPT	Three-phase inverter	AGPU, SPT, Three-phase inverter
IC (A)	2.1	2.1	2.1
V (V)	3.0	2.3	2.4
R_ce_(on) (mΩ)	33	33	356
Turn-on energy ET, on (mJ)	14.6	1.1	4
Turn-off energy ET, off (mJ)	6.5	0.8	16

**Table 9:** Specification of the SiC-based semiconductor device.

Parameter	Unit	3C-SiC BJT	6H-SiC JFET	4H-SiC SBD	4H-SiC MOSFET
Bandgap	eV	2.2	3.0	3.26	3.26
Critical electric field	Mv/cm	1.5	3	3	3
Electron mobility	cm^2^/V-s	1000	370	800	800
Saturated electron drift velocity	cm/s	2e6	2e6	2e6	2e6
Thermal conductivity	W/cm-K	4.9	4.9	4.9	4.9
Lattice mismatch	%	3.5	3.5	3.5	3.5
Wafer size	mm	100	150	150	150
Voltage rating	kV	1.2	1.7	1.7	1.7
Current rating	A	15	10	24	24
Switching frequency	Hz	10	50	-	100
Resistivity	Ω-cm	10^4^	10^4^	10^4^	10^4^
Channel resistance	Ω-cm^2^	120	35	-	80
Stress levels of voltage	V	5000	10,000	10,000	10,000
Stress levels of current	A	15	10	24	24
Off-state breakdown voltage	kV	1.2	1.7	1.7	1.7
Maximum junction temperature	°C	500	500	600	600
Temperature range	°C	−55 to 250	−55 to 250	−55 to 300	−55 to 300
Temperature stability	°C	1	1	1	1
Conduction losses		Medium	Low	Low	Low
Switching losses		Medium	Medium	-	High
Power losses		Medium	Medium	Low	Medium
Baliga’s figure of merit		51	204	408	408
Johnson’s figure of merit		8	31	62	62
Applications		Medium voltage, medium frequency	High voltage, high frequency	High voltage rectifier	High voltage, high frequency

**Table 10:** Comparison between Si-based and SiC-based generator controller [115,116].

Parameters	Unit	Si-Based Generator Controller	SiC-Based Generator Controller
Output power	kW	100	200
Aux SSPC channels	kW	No (Excitation Controller)	120 (2ea 600 V × 100 A)
Bi-directional	-	Yes	Yes
Space claim (volume)	L	33.6	17.4
Size	mm	444 × 400 × 189	350 × 350 × 142
Power density (size)	kW/L	3	12
Weight	Kg	27.7	25
Power density (weight)	kW/kg	3.6	8
Coolant temp, max	°C	85	105
Ambient temp, max	°C	71	121
Communications	-	IEEE 1394B Bus	CANBus

**Table 11:** SiC power module by GE Aerospace [117].

Type of SiC Module	Part Number	Voltage Rating (V)	Current Rating (A)	R_DS_(on) @25 °C (mΩ)	Thermal Cooling System (R_th(j-c)_) (K/W)	Size (Width × Length) (mm)	Maximum Junction Temp (°C)
Half-bridge	GE12047CCA3	1200	475	3.1	0.1	48 × 86	175
	GE17042CCA3	1700	425	3.8			
	GE12090CDA3	1200	875	1.6	0.03	100 × 140	
	GE17080CDA3	1700	765	1.9			
	GE12160CEA3	1200	1425	1.0	0.03	90 × 134	
	GE17140CEA3	1700	1275	1.2			
Dual-bridge	GE12047BCA3	1200	475	3.1	0.1	48 × 86	
	GE17042BCA3	1700	425	3.8			
6-switch	GE12050HEA3	1200	6 × 475	3.1	0.1	90 × 134	
	GE17045HEA3	1700	6 × 425	4.8			
6-pack	GE12050EEA3	1200	3 × 475	3.1	0.1		
	GE17045EEA3	1700	3 × 425	3.8			

**Table 12:** Evaluation of power electronic GaN-based semiconductor device.

Year	Device	Specifications	Milestone	Features
1990s	HEMT	50 V, 1 A	First GaN transistor	Higher frequency than Si and GaAs
2000s	HEMT	200 V, 1 A	Enhancement mode GaN	Normally off operation
2010s	HEMT	600 V, 30 A	High-voltage GaN transistors	Replacing Si MOSFETs in adapters
	MISFET	200 V, 1 A	GaN MISFETs	Gate oxide reliability improvements
2015	HEMT	1200 V, 15 A	>1 kV rating achieved	Entering high-voltage applications
2019	MISFET	650 V, 20 A	Commercial GaN MISFETs	Reduced gate leakage over HEMTs
2020s	HEMT	3.3 kV+, 100 A+	High current density	Targeting EV traction inverters
	MISFET	3.3 kV+, high current	Further MISFET refinement	Improved reliability over HEMTs
Future	IGBT	1.2 kV+, high current	GaN on Si IGBTs	Improving Si substrates and vertical GaN
	Thyristor	Medium voltage, high current	GaN thyristors	High-current applications

**Table 13:** GaN rectifier diode specification by Avogy, now known as Nexgen Power Systems [166].

Model	Type	URRM/V	If/A	IR/μA	QC/nC
AVDO2A600A	SBD	600	2	150	4
AVDO5A120A	PN	1200	5	0.1	7
AVDO5A170A	PN	1700	5	0.1	14

**Table 14:** Characteristics of commercial GaN MOSFETs [28].

Part Number	Current (A)	C_ISS_ (pF)	C_GD_ (pF)	R_ds_(on) @25 °C (Ω)
TP65H150G4PS	2.5	307	1	0.15
TP65H150G4PS	5	307	1	0.15
TP65H150G4PS	10	307	1	0.15
TP65H070L	20	600	4	0.072
TP65H035WSQA	40	1500	14	0.035

**Table 15:** Specification of the different GaN-based semiconductor devices.

Parameter	Unit	GaN HEMT	GaN MISFET	GaN SBD
Bandgap	eV	3.4	3.4	3.4
Critical electric field	Mv/cm	3.3	3.3	3.3
Electron mobility	cm^2^/V-s	2000	1500	2000
Saturated electron drift velocity	cm/s	1 × 10^5^	1 × 10^5^	1 × 10^5^
Thermal conductivity	W/cm-K	1.3	1.3	1.3
Lattice mismatch	%	15	15	15
Wafer size	mm	150	150	150
Voltage rating	kV	1.2	1	1.2
Current rating	A	30	20	30
Switching frequency	Hz	1000	500	-
Resistivity	Ω-cm	106	106	106
Channel resistance	Ω-cm^2^	8	10	-
Stress levels of voltage	V	600	500	600
Stress levels of current	A	30	20	30
Off-state breakdown voltage	kV	1.2	1	1.2
Maximum junction temperature	°C	250	250	250
Temperature range	°C	−55 to 250	−55 to 250	−55 to 250
Temperature stability	°C	-	-	-
Conduction losses		Medium	Medium	Low
Switching losses		Low	Medium	-
Power losses		Medium	Medium	Low
Baliga’s figure of merit		26	26	26
Johnson’s figure of merit		46	23	46
Applications		High frequency	Medium frequency	Rectifier

**Table 16:** Most popular GaN driver solutions [31].

Manufacturer	Model	Split Outputs	Bootstrap Voltage Management	Configuration	Features
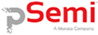	PE29101	Yes	Yes	Half-bridge	Frequency < 33 MHz
	PE29102	Yes	No	Half-bridge	Frequency < 33 MHz
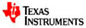	LMG1205	Yes	Yes	Half-bridge	Automotive-qualified
	LM5113-Q1	Yes	Yes	Half-bridge	General purpose
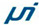	uP1966A	Yes	Yes	Half-bridge	General purpose

**Table 17:** Evaluation of diamond-based semiconductor device.

Year	Device	Specifications	Milestone	Features
1980s	Schottky diode	Single-crystal research only	First diamond electronics	Extremely high bandgap
1990s	Schottky diode	Single-crystal, <100 V	Small single-crystal diodes	High-temperature operation
2000s	Schottky diode	Polycrystalline, 400 V	First polycrystalline devices	Manufacturable on poly diamond films
2010s	Vertical JFET	Polycrystalline, 50 V	First diamond vertical transistors	Blocking voltage and operating temperature increase
2018	Lateral MOSFET	Polycrystalline, 200 V	Diamond lateral MOSFET	High current density demonstrated
2020s	Vertical MOSFET	Polycrystalline, >1 kV	Diamond vertical power MOSFETs	Targeting commercial viability
Future	Bipolar transistor	Polycrystalline, >1 kV	High-voltage diamond bipolar transistors	Complementing MOSFETs
	Thyristor	>10 kV blocking voltage	Ultra-high voltage rectifiers and switches	Surpassing limitations of existing technologies
	IGBT	>10 kV, high current density	Diamond IGBTs	Theoretical capabilities unmatched by any material

**Table 18:** Specification of diamond-based semiconductor device.

Parameter	Unit	Diamond Bipolar Transistor	Diamond Schottky Diode	Diamond MISFET
Bandgap	eV	5.45	5.45	5.45
Critical electric field	Mv/cm	10	10	10
Electron mobility	cm^2^/V-s	1800	2200	1800
Saturated electron drift velocity	cm/s	2.7 × 10^7^	2.7 × 10^7^	2.7 × 10^7^
Thermal conductivity	W/cm-K	22	22	22
Voltage rating	kV	>10	>10	>10
Current rating	A	5	10	5
Switching frequency	Hz	>1000	-	>1000
Resistivity	Ω-cm	>1011	>1011	>1011
Channel resistance	Ω-cm^2^	<1	-	<1
Stress levels of voltage	V	>10,000	>10,000	>10,000
Stress levels of current	A	5	10	5
Off-state breakdown voltage	kV	>10	>10	>10
Maximum junction temperature	°C	>600	>600	>600
Temperature range	°C	−55 to 250	−55 to 250	−55 to 250
Conduction losses		Very Low	Very Low	Very low
Switching losses		Low	-	Low
Power losses		Very Low	Very Low	Very low
Baliga’s figure of merit		1650	1650	1650
Johnson’s figure of merit		288	288	288
Applications		High power, high frequency	Rectifier	High power, high frequency

**Table 19:** Comparisons of the specifications of Si, SiC, GaN, and diamond.

Parameter	Unit	Si	SiC	GaN	Diamond
Bandgap	eV	1.1	3.0–3.4	3.4–3.6	5.45
Critical electric field	Mv/cm	0.3	3	3.3	10
Electron mobility	cm^2^/V-s	1500 to 2000	100 to 600	1000 to 2000	2200
Saturated electron drift velocity	cm/s	10^5^	2 × 10^6^	10^5^	2.7 × 10^7^
Thermal conductivity	W/cm-K	1.5 to 2.0	3.0 to 4.9	1.0 to 1.5	22
Lattice mismatch	%	-	3.5	15	-
Wafer size	mm	300	150	150	-
Voltage rating	kV	<1	10	1.2	>10
Current rating	A	100	20	30	10
Switching frequency	Hz	20 k	400 k	1 M	>1 M
Power density (size-wise)	W/cm^2^	~5	~10	~30	>100
Power density (weight-wise)	W/g	~5	~10	~20	>100
Resistivity	Ω-cm	10	104	106	>1011
Channel resistance	Ω-cm^2^	30	5	8	<1
Stress levels of voltage	V	600	10,000	600	>10,000
Stress levels of current	A	100	100	30	10
Off-state breakdown voltage	kV	0.6	10	1.2	>10
Maximum junction temperature	°C	150	600	250	>600
Temperature range	°C	−55 to 200	−55 to 300	−55 to 250	−55 to 250
Temperature stability	°C	-	1	-	-
Conduction losses		High	Low	Medium	Very Low
Switching losses		Low	High	Medium	Low
Power losses		High	Medium	Medium	Very Low
Baliga’s figure of merit		1	408	26	1650
Johnson’s figure of merit		1	62	46	288
Applications		Low voltage low power	High voltage, high power	High frequency	High power, high frequency

**Table 20:** Material characteristics of WBG and UWBG semiconductors [17].

Parameter	Unit	WBG		UWBG		
		GaN	4H-SiC	AlGaN/AlN	β-Ga_2_O_3_	Diamond
Bandgap	eV	3.4	3.3	Up to 6.0	4.9	5.5
Thermal conductivity (Ԑ)	Wm^−1^K^−1^	253	370	253–319	11–27	2290–3450
Substrate quality (dislocations)	per cm^2^	≈10^4^	≈10^2^	≈10^4^	≈10^4^	≈10^5^
Substrate diameter	inch	8 (on Si)	8	2	4	1
Demonstrated p-type dopability	-	Good	Good	Poor	No	Good
Demonstrated p-type dopability	-	Good	Good	Moderate	Moderate	Moderate

**Table 21:** State-of-the-art performance ranges for the evaluated powers and frequencies [28].

P (kW)	Si	SiC	GaN
1	-	Up to 14 kHz	14–500 kHz
2	-	Up to 28 kHz	28–500 kHz
4	-	Up to 55 kHz	55–500 kHz
8	-	Up to 110 kHz	110–500 kHz
16	-	1–500 kHz	-

**Table 22:** Figure-of-merit comparison of Si, SIC, GaN, and diamond materials [214].

Parameter	Unit	Si	4H-SiC	Wurtzite GaN	Diamond
Electron mobility (µ)	cm^2^/V-s	1360	700	1500	2200
Relative dielectric constant (Ԑ)	-	11.7	9.7	8.9	5.7
Circuit electric field (Ec)	MV/cm	0.3	2.2	3.3	20
Thermal conductivity (σth)	W/cm-K	1.3	3.7	13.3	20
HMFOM	Ec √µ	1	5.26	11.55	84.79
HCAFOM	Ԑ E^2^c √µ	1	31.99	96.66	2753.9
HTFOM	σth/Ԑ *Ec*	1	0.47	0.12	0.47

**Table 23:** A device level comparison among three unipolar power transistors. Si MOSFET = IXTH30N60P, SJ MOSFET = IPD65R225C7, SJ MOSFET = C3M0280090D, GaN HFET = GS66504B estimated die size [220].

Parameter	Unit	Si MOSFET	Si-SJ MOSFET	SiC MOSFET	GaN HEFT
Breakdown voltage	V	600	650	900	600
Breakdown current	A	30	11	11.5	15
Wafer size	mm	-	200	100	150
Die area	mm^2^	41	6.6	2.1	6.5
Current density	A/cm^2^	-	170	540	230
R_on_	Ω	0.24	0.22	0.28	0.11
DFOM1 (R_on_ × Q_g_)	Ω-nC	19.68	4.4	2.66	0.11
DFOM2 (R_on_ × Q_gd_)	Ω-nC	7.2	1.32	0.95	0.11
DFOM3 (R_on_ × Q_oss_)	Ω-nC	48.9	83.7	6.93	3.08
DFOM4 (R_on_ × Q_rr_)	Ω-nC	960	1320	13.16	2.8
Rjc	°C/W	0.23	1.99	2.3	1
Normalized die cost	σth/Ԑ *Ec*	-	1	4	3.6

**Table 24:** Features and values comparison among Si, SiC, GaN, and diamond.

Material	Device	Voltage (V)	Current (A)	Frequency (kHz)	Applications	Package	Features	Manufacturer
Si	IGBT	6500	1200	20	Motor drives, power supplies, converters	Discrete, Module	High power capability, easy to parallel	Infineon, STMicroelectronics
	MOSFET	900	150	100	Switching power supplies, motor drives	Discrete, Module	Fast switching, low losses	Infineon, ON Semiconductor
SiC	MOSFET	1700	100	100	EV drivetrains, PV inverters, power supplies	Discrete, Module	High efficiency, high frequency, high temperature	Wolfspeed, Rohm
	SBD	1700	20	-	HVDC, motor drives, battery charging	Discrete	Low loss rectification, fast recovery	Wolfspeed, Infineon
GaN	HEMT	1200	15	1000	Adapters, data center power, wireless power	Discrete, Module	High frequency, high efficiency	Efficient Power Conversion, Navitas
	MISFET	650	20	500	On-board chargers, power supplies, DC–DC converters	Discrete	Normally off, low losses	Transphorm, Panasonic
Diamond	Schottky Diode	400	0.1	-	High-temperature electronics	Research	Extremely high-temperature capability	Akhan Semiconductor
	JFET	50	0.1	-	High-temperature electronics	Research	High bandgap, temperature tolerance	Group4 Labs

**Table 25:** Current commercial semiconductor devices and their ratings.

Manufacturing Company	Device Type	Part Number	Voltage Rating (V)	Current Rating (A)	Switching Frequency (Hz)	Operating Temp. (°C)
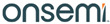	Si Diode [241]	NXH80B120L2Q0SNG	1000 to 1200	30 to 400	10 k	−40 to 175
	SiC Diode [242]	FFSM1065A	650 to 1700	4 to 20	-	−55 to 175
	SiC MOSFET [243,244]	NVBG095N065SC1, NTH4L028N170M1	650 to 1700	30 to 81	1 M	14 to 175
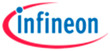	GaN Transistor [245,246]	IGOT60R042D1, IGLD60R070D1	400 to 600	60 to 12.2	100 k	−55 to 150
	Si Diode [247,248]	IDW100E60, IDP30E120XKSA1	600 to 1200	28 to 150	18 k to 100 k	−55 to 150
	SiC MOSFET [205,249]	IMBG65R022M1H, DF419MR20W3M1HFB11	650 to 2000	50 to 60	_	−55 to 150
	Automotive IGBT [250,251]	F450R07W1H3B11A, FS380R12A6T4B	650 to 1200	50 to 380	2 k to 50 k	−40 to 125
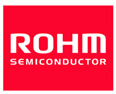	GaN HEMT [252]	GNE1040TB	150 to 150	10 to 20	-	−55 to 150
	SiC MOSFET [253,254]	SCT3017AL, SCT3017ALHR, SCT2H12NY	650 to 1700	21 to 04	1 M	−55 to 175
	Si MOSFET [255,256]	HP8JE5, HP8KC6, HP8MB5, UT6MA3	20 to 40	4.5 to 5.5	_	−55 to 150
	Ignition IGBT [257]	RGPR10BM40FH, RGPR20NL43HR	400–430	20 to 30	_	−55 to 175
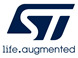 ST Microelectronics	SiC Diode [258,259]	STPSC2006CW, STPSC40H12C	600 to 1200	10 to 40	1 M	−40 to 175
	SiC MOSFET [112,260]	SCT1000N170	650 to 1700	7 to 300	12 k to 25 k	−55 to 200
	GaN HEMT [261,262]	MASTERGAN1, MASTERGAN3	600 to 650	10 to 10.5	500 k to 2 M	−40 to 150
	IGBTs [263]	STG15M120F3D7, STG200G65FD8AG	300 to 1700	10 to 200	1 M	−55 to 150

## Data Availability

Not applicable.
